# Novel Drivers of Virulence in Clostridioides difficile Identified via Context-Specific Metabolic Network Analysis

**DOI:** 10.1128/mSystems.00919-21

**Published:** 2021-10-05

**Authors:** Matthew L. Jenior, Jhansi L. Leslie, Deborah A. Powers, Elizabeth M. Garrett, Kimberly A. Walker, Mary E. Dickenson, William A. Petri, Rita Tamayo, Jason A. Papin

**Affiliations:** a Department of Biomedical Engineering, University of Virginia, Charlottesville, Virginia, USA; b Department of Medicine, Division of Infectious Diseases & International Health, University of Virginia, Charlottesville, Virginia, USA; c Department of Biochemistry & Molecular Genetics, University of Virginia, Charlottesville, Virginia, USA; d Department of Microbiology, Immunology and Cancer Biology, University of Virginia Health System, Charlottesville, Virginia, USA; e Department of Pathology, University of Virginia Health System, Charlottesville, Virginia, USA; f Department of Microbiology & Immunology, University of North Carolina Chapel Hill School of Medicine, Chapel Hill, North Carolina, USA; Mayo Clinic

**Keywords:** *Clostridioides difficile*, metabolic modeling, transcriptomics, virulence factors

## Abstract

The pathogen Clostridioides difficile causes toxin-mediated diarrhea and is the leading cause of hospital-acquired infection in the United States. Due to growing antibiotic resistance and recurrent infection, targeting C. difficile metabolism presents a new approach to combat this infection. Genome-scale metabolic network reconstructions (GENREs) have been used to identify therapeutic targets and uncover properties that determine cellular behaviors. Thus, we constructed C. difficile GENREs for a hypervirulent isolate (strain [str.] R20291) and a historic strain (str. 630), validating both with *in vitro* and *in vivo* data sets. Growth simulations revealed significant correlations with measured carbon source usage (positive predictive value [PPV] ≥ 92.7%), and single-gene deletion analysis showed >89.0% accuracy. Next, we utilized each GENRE to identify metabolic drivers of both sporulation and biofilm formation. Through contextualization of each model using transcriptomes generated from *in vitro* and infection conditions, we discovered reliance on the pentose phosphate pathway as well as increased usage of cytidine and *N*-acetylneuraminate when virulence expression is reduced, which was subsequently supported experimentally. Our results highlight the ability of GENREs to identify novel metabolite signals in higher-order phenotypes like bacterial pathogenesis.

**IMPORTANCE** Clostridioides difficile has become the leading single cause of hospital-acquired infections. Numerous studies have demonstrated the importance of specific metabolic pathways in aspects of C. difficile pathophysiology, from initial colonization to regulation of virulence factors. In the past, genome-scale metabolic network reconstruction (GENRE) analysis of bacteria has enabled systematic investigation of the genetic and metabolic properties that contribute to downstream virulence phenotypes. With this in mind, we generated and extensively curated C. difficile GENREs for both a well-studied laboratory strain (str. 630) and a more recently characterized hypervirulent isolate (str. R20291). *In silico* validation of both GENREs revealed high degrees of agreement with experimental gene essentiality and carbon source utilization data sets. Subsequent exploration of context-specific metabolism during both *in vitro* growth and infection revealed consistent patterns of metabolism which corresponded with experimentally measured increases in virulence factor expression. Our results support that differential C. difficile virulence is associated with distinct metabolic programs related to use of carbon sources and provide a platform for identification of novel therapeutic targets.

## INTRODUCTION

Clostridioides difficile is a Gram-positive, sporulating anaerobe that remains a critical problem in health care facilities across the developed world (1, 2). Susceptibility to C. difficile infection (CDI) is most frequently preceded by exposure to antibiotic therapy (3). While these drugs are lifesaving, they also diminish the abundance of other bacteria in the microbiota, altering the metabolic environment of the gut and leaving it susceptible to colonization by C. difficile (4–6). Recently, we observed that C. difficile adapts transcription of distinct catabolic pathways to the unique conditions in susceptible gut environments following different antibiotic pretreatments (7, 8). These transcriptional shifts indicated that C. difficile must coordinate metabolic activity accordingly to compete within new hosts. In spite of these differences, there are known core elements of C. difficile metabolism across different environments including carbohydrate and amino acid fermentation (9). It is known that specific growth nutrients influence expression of virulence genes in C. difficile (9, 10). Given these findings, targeted therapeutic strategies that alter active metabolism and downregulate virulence may be possible without continued exposure to antibiotics. This form of treatment would be especially beneficial as there have been stark increases in the prevalence of antibiotic resistance and hypervirulence among C. difficile clinical isolates (11, 12).

Genome-scale metabolic network reconstructions (GENREs) are mathematical formalizations of metabolic reactions encoded in the genome of an organism. These models are subsequently constrained by known biological and physical parameters such as membrane transport and enzyme kinetics. GENREs can be utilized to interrogate the metabolic capability of a given organism, as well as providing a means to simulate growth and assess the impact of genotype on metabolism. GENREs have been implemented in directing genetic engineering efforts (13) and accurately predicting auxotrophies and interactions between species for growth substrates (14, 15). These platforms also create improved context for the interpretation of omics data (16), and have provided powerful utility for identification of novel drug and gene targets, accelerating downstream laboratory testing (17). This concept is especially critical when delineating a complex array of signals from communities of organisms like the gut microbiome (18). Leveraging these tools, several recent studies have identified nodes of metabolism that promise to provide novel therapeutic targets in clinically relevant pathogens including Klebsiella pneumoniae, Staphylococcus aureus, and Streptococcus mutans (17, 19, 20). However, there has been limited progress to date applying GENREs to obtain mechanistic understanding for metabolism during infection as they relate to colonization and virulence. Taken together, these principles make GENREs strong platforms for deciphering novel metabolic drivers of virulence-associated phenotypes in C. difficile.

We began by generating new GENREs for two strains of C. difficile including a highly characterized laboratory strain, C. difficile strain (str.) 630 (21), as well as a more recently isolated hypervirulent strain, R20291 (22). *De novo* reconstruction for both models was followed by extensive literature-driven manual curation of catabolic pathways and related metabolite transport, with specific emphasis on Stickland fermentation for ATP generation and C. difficile-specific redox maintenance (23). Additionally, both GENREs contain a tailored biomass objective function (an *in silico* proxy for bacterial growth, requiring synthesis of major macromolecular components) which accounts for codon biases and amino acid balance and cell wall structure. Growth simulations from both GENREs were compared against *in vitro* gene essentiality and carbon utilization screens, which indicated significant levels of agreement across all validation data sets.

To assess potential mechanisms of metabolic control of virulence, we then created context-specific models of C. difficile metabolism by integrating transcriptomic data collected from both laboratory culture and infection conditions where differential expression of C. difficile virulence factors was observed. Overall, during increased virulence expression both strains of C. difficile were predicted to favor increased fermentation of amino acids and decreased reliance on carbohydrate usage. Specifically in the hypervirulent strain R20291 during states of phase variation, we found efflux of the biofilm component *N*-acetylglucosamine in variants known to produce significantly more biofilm experimentally. Additionally, this state was predicted to have increased reliance on glucose to fuel nucleotide synthesis, instead of ATP generation. When tested *in vitro*, we indeed found that the colony morphology associated with this phase variant was dependent on environmental glucose availability. Alternatively, in infection-specific models of strain 630, we identified consistent patterns of proline and ornithine fermentation in states of both high and low sporulation, which agreed with metabolomic analysis of each condition. However, in instances of lower spore burden our model predicted significantly greater usage of the host-derived glycan *N*-acetylneuraminate (Neu5Ac) and the nucleotide precursor cytidine as primary sources of carbon. In subsequent laboratory testing we were able to show that not only can C. difficile use the substrates for growth but also both lead to lower quantities of spores, which are essential for transmission of the pathogen (24, 25). This work is the first time that contextualized GENREs of a pathogen have been utilized to identify new metabolite signals of virulence regulation. As such, the high-quality GENREs described here can greatly augment the discovery of novel metabolism-directed therapeutics to treat CDI. Moreover, our results demonstrate that GENREs provide an advantage for delineating complex patterns in transcriptomic and metabolomic data sets into tractable experimental targets.

## RESULTS

### C. difficile metabolic network generation, gap-filling, and curation.

The emergence of hypervirulent strains of C. difficile that have unique metabolism and virulence factors highlights the importance for the in-depth study of metabolic pathways to understand and identify targets within these isolates. Core metabolic processes also present an attractive target for novel antimicrobial measures as they may be less likely to allow for acquired antibiotic resistance (26). With these concepts in mind, we focused on the best-characterized hypervirulent isolate, str. R20291. However, to maximize the utility of the bulk of published C. difficile metabolic research, we elected to generate a reconstruction for the lab-adapted str. 630 in parallel. This focus afforded the ability to continuously cross-reference curations between the models and to more readily identify emergent differences that are specifically due to genomic content.

We began the reconstruction process by accessing the reannotated genome of str. 630 (27) and the published str. R20291 genome (22), both available on the Pathosystems Resource Integration Center database (PATRIC) (28). Following an established protocol for creating high-quality genome-scale models (29), and utilizing the ModelSEED framework and modified reaction database (30), we created scaffold reconstructions for both strains. We subsequently performed complete translated proteome alignment between str. 630 and str. R20291, resulting in 684 homologous metabolic gene products and 22 and 33 unique gene products, respectively (see Table S2 in the supplemental material). Among the distinctive features were additional genes for dipeptide import in str. 630 and glycogen import and utilization in str. R20291, which have both been linked to modulated levels of virulence across strains of C. difficile (31, 32).

10.1128/mSystems.00919-21.7 TABLE S2 C. difficile 630 and R20291 PATRIC protein sequence alignment results. Download Table S2, XLSX file, 0.3 MB. Copyright © 2021 Jenior et al. 2021 Jenior et al. https://creativecommons.org/licenses/by/4.0/ This content is distributed under the terms of the Creative Commons Attribution 4.0 International license.

Manual curation is required to ultimately produce high-quality GENREs and make meaningful biological predictions (33). As such, we proceeded to manually incorporate 259 new reactions (with associated genes and metabolites) and altered the conditions of an additional 312 reactions already present within each GENRE prior to gap-filling (Table S1). Primary targets and considerations for the manual curation of the C. difficile GENREs included the following:

- Anaerobic glycolysis, fragmented tricarboxylic acid (TCA) cycle, and known molecular oxygen detoxification (23, 34)
- Minimal medium components and known auxotrophies (35–37)
- Aminoglycan and dipeptide catabolism (38–40)
- Numerous Stickland fermentation oxidative and reductive pathways (Table S2) (41–52)
- Carbohydrate fermentation and short-chain fatty acid metabolism (41, 53–55)
- Elements of the Wood-Ljungdahl pathway (56)
- Energy metabolite reversibility (e.g., ATP, GTP, FAD, etc.) (57)
- Structural components including teichoic acid, peptidoglycan, and isoprenoid biosynthesis
- Additional pathogenicity-associated metabolites (e.g., *p*-cresol [44] and ethanolamine [58])

10.1128/mSystems.00919-21.6 TABLE S1 GENRE creation steps, biomass formulation, gap-filling medium compositions, and GENRE statistics. Download Table S1, XLSX file, 0.03 MB. Copyright © 2021 Jenior et al. 2021 Jenior et al. https://creativecommons.org/licenses/by/4.0/ This content is distributed under the terms of the Creative Commons Attribution 4.0 International license.

Following the outlined manual additions, we created a customized biomass objective function with certain elements tailored to each strain of C. difficile and complete synthesis requirements for macromolecular components. Our biomass objective function formulation was initially adapted from the well-curated GENRE of the close phylogenetic relative Clostridium acetobutylicum (59) with additional considerations for tRNA synthesis and formation of cell wall macromolecules, including teichoic acid and peptidoglycan (Table S1). Coefficients within the formulations of DNA replication, RNA replication, and protein synthesis component reactions were adjusted by genomic nucleotide abundances and codon frequencies to yield strain-specific biomass objective functions (60). To successfully simulate growth, we next performed an ensemble-based parsimonious flux balance analysis (pFBA) gap-filling approach (61, 62), utilizing a metabolic reaction database centered on Gram-positive anaerobic bacterial metabolism (see Materials and Methods). Gap-filling refers to the automated process of identifying incomplete metabolic pathways due to an absence of genetic evidence that are necessary for *in silico* growth and addition of the minimal functionality needed to achieve flux through these pathways (63). We performed gap-filling across six distinct and progressively more limited medium conditions—complete medium, brain heart infusion (BHI) (64), C. difficile defined medium with and without glucose (CDM) (37), no-carbohydrate minimal medium (NCMM) (5), and basal defined medium (BDM) (35)—which added a total of 68 new reactions that allowed for robust growth across all conditions, the largest fraction of which was involved in large phospholipid biosynthesis for generation of more complex cell wall components (Table S1).

The final steps of the curation process were focused on limiting the directionality of reactions known to be irreversible, extensive balancing of the remaining incorrect reaction stoichiometries, and adding annotation data for all network components. This step also included adding gene associations to gap-filled reactions where possible. After completion, we repeated the assessments that were performed for the earlier reconstructions and found that our GENREs had substantial improvements in all metrics including few, if any, flux or mass inconsistencies, and now each received a cumulative MEMOTE score of 86% (Table S1). The new network reconstructions were designated iCdG709 (str. 630) and iCdR703 (str. R20291).

### C. difficile GENRE validation against laboratory measurements.

A standard measurement of GENRE performance is the comparison of predicted essential genes for growth *in silico* and those found to be essential experimentally through forward genetic screens (65). For a gene to be considered essential, less than 1% of optimal biomass can be produced by a given mutant (the equivalent of no observable growth) during single-gene knockout simulations (66). Recently, a large-scale transposon mutagenesis screen was published for str. R20291 (67), and as such, we utilized the proteomic alignment from the previous section to determine homologs in str. 630. While interpretation of results from *in vitro* essentiality screens of this nature can be difficult and associated with a degree of noise, they provide an important starting point for initial model validation as well as a basis for future curation efforts. Simulating growth in BHI rich medium, we identified essential genes for both models, which revealed overall accuracies of 89.1% and 88.9%, with negative predictive values as high as 90.0% for iCdR703 and 89.9% for iCdG709 (Fig. S1A). This high degree of agreement supported that metabolic pathways in the new GENREs were structured correctly and are more likely to provide useful downstream predictions.

**FIG 1:**
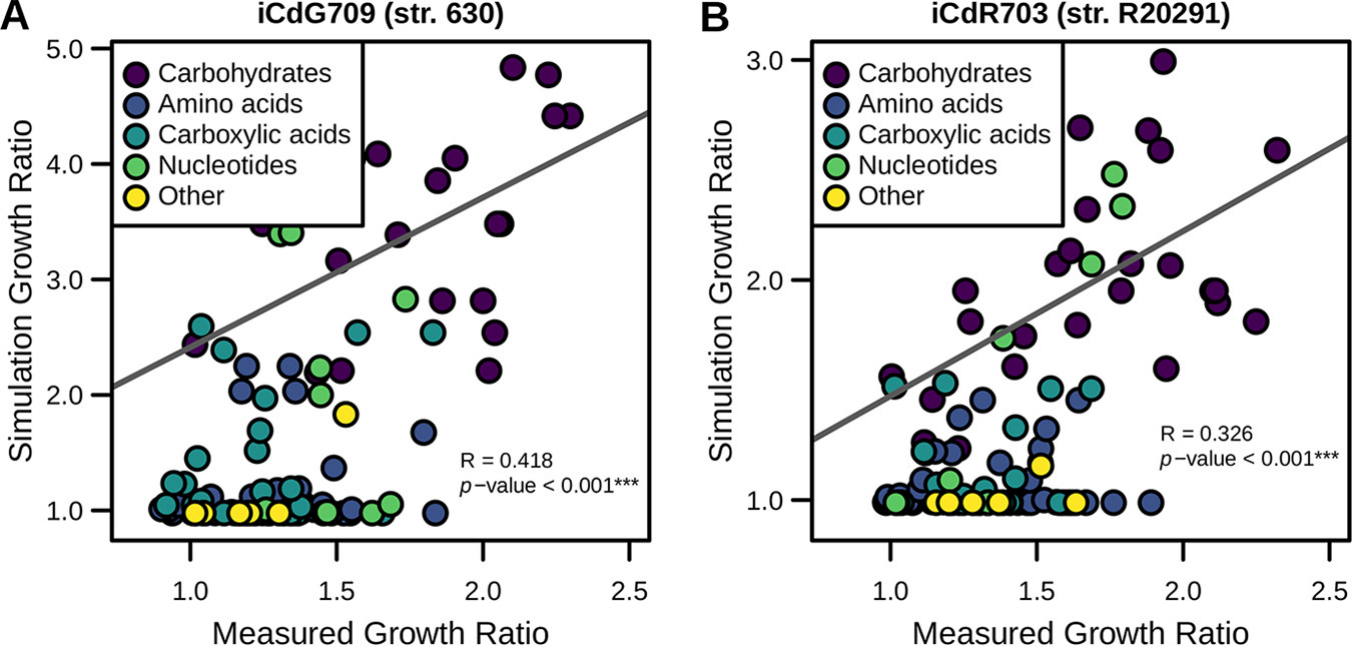
Carbon source utilization prediction profiles accurately reflect laboratory measurements. Results from previous phenotypic screen of 115 metabolites for both str. 630 and str. R20291 were compared against *in silico* results for each corresponding GENRE. Ratios of overall *in vitro* growth enhancement by each metabolite were correlated with the corresponding results from growth simulations in the same media for iCdG709 (str. 630) (A) and iCdR703 (str. R20291) (B). Points are colored by their biochemical grouping, fit, and significance determined by Spearman correlation.

10.1128/mSystems.00919-21.1 FIG S1 Additional C. difficile GENRE validation against laboratory measurements. (A) *In silico* gene essentiality predictions for both GENREs cross-referenced with the Dembek et al. (M. Dembek, L. Barquist, C. J. Boinett, A. K. Cain, et al., mBio 6:e02383-14, 2015, https://doi.org/10.1128/mbio.02383-14) transposon screen for str. R20291 (iCdG709 [str. 630] utilizes homologs from the genome of str. R20291). (B) Binary quantification for metabolite growth enhancement shown for both strains/GENREs in Fig. 1. Positive predictive values were 95.1% for iCdR703 and 92.3% for iCdG703. (C) Computationally determined minimum growth substrates for both GENREs compared with experimentally determined C. difficile minimal medium components across three previously published studies. Essentiality was determined for those genes and metabolites that when absent resulted in a yield of <1.0% of optimal biomass flux during growth simulation utilizing components of the corresponding media used experimentally. Download FIG S1, PDF file, 0.4 MB. Copyright © 2021 Jenior et al. 2021 Jenior et al. https://creativecommons.org/licenses/by/4.0/ This content is distributed under the terms of the Creative Commons Attribution 4.0 International license.

To then assess if GENRE requirements reflected the components of minimal medium derived experimentally, we identified the minimum subset of metabolites necessary for growth. Through systematic limitation of extracellular metabolites, we were able to determine the impact of each component on achieving some level of biomass flux (Fig. S1C). This analysis revealed that most metabolites found to be essential during growth simulation have also been shown experimentally to be required for *in vitro* growth in basal defined medium (BDM) (35). Interestingly, while growth simulations indicated that neither iCdG709 (str. 630) nor iCdR703 (str. R20291) was auxotrophic for methionine, the published formulation of BDM indicates methionine is found to be largely growth enhancing but not essential for low levels of growth (36). Additionally, it has been demonstrated in the laboratory that C. difficile is able to harvest sufficient bioavailable sulfur from excess cysteine instead of methionine (37, 68), further supporting growth simulation results. Similarly, the published formulation of BDM indicates that pantothenate (vitamin B_5_) appears to enhance growth rate only *in vitro* and is not necessarily required to support low growth rates. Our results also indicated that iCdR703 was not auxotrophic for isoleucine relative to iCdG709 and indeed contained additional genes coding for synthesis of a precursor (3S)-3-methyl-2-oxopentanoate (*ilvC*, a ketol-acid reductoisomerase) which were not present in its counterpart GENRE (Table S2). In summary, the *in silico* minimal requirements for iCdG709 and iCdR703 closely mirrored experimental results for both strains of C. difficile in the laboratory.

We next assessed additional carbon sources that impact the growth yield predictions for both GENREs. Utilizing previously published results for both C. difficile strains in a high-throughput screen (69), we simulated growth for each carbon source individually in background minimal medium and calculated the shift in optimal growth rate. Importantly, C. difficile is auxotrophic for specific amino acids (e.g., proline [Fig. S1C]) that it is also able to catabolize through Stickland fermentation (70), so the background medium must be supplemented with low concentrations of each. As such, the values are reported as the ratio of the final optical density for growth with the given metabolite to low levels of growth observed in the background medium alone. Through correlation of the results from these two comparisons, we were able to assess how well *in silico* predictions matched experimental results. Across all the 116 total metabolites that were in both the *in vitro* screen and the C. difficile GENREs, we identified significant predictive correlations in the amount of growth enhancement for iCdG709 and iCdR703 (*P* values < 0.001) (Fig. 1A and B). This relationship was even more pronounced for carbohydrates and amino acids, the primary carbon sources for C. difficile. When these predictions were reduced to binary interpretations of either enhancement or nonenhancement of growth, we found that iCdG709 predicted 92.8% and iCdR703 predicted 96.6% true-positive enhancement calls (Fig. S1B). This metric is most valuable here as it indicates that each GENRE possesses the necessary machinery for catabolizing a given metabolite. Collectively, these data strongly indicated that both GENREs were well suited for prediction of growth substrate utilization in either strain of C. difficile.

Finally, we also compared our results against existing C. difficile GENREs. The primary focus of curated C. difficile metabolic modeling efforts has been on the first fully sequenced strain of C. difficile, str. 630. The first reconstruction effort (iMLTC806cdf [71]) and subsequent revision (icdf834 [71, 72]) were followed by a recent *de novo* creation following updated genome curation (iCN900 [27, 73). Another GENRE was developed for str. 630Δerm (iHD992 [74]), a strain derived from str. 630 by serial passage until erythromycin resistance was lost (75). Four additional C. difficile strain GENREs were generated as a part of an effort to generate numerous new reconstructions for members of the gut microbiota (76); these reconstructions received only semiautomated curation performed without C. difficile-specific considerations. To establish a baseline for the metabolic predictions possible with current C. difficile GENREs, we selected common criteria with large impacts on the quality of subsequent predictions for model performance (Table S3). The first of these metrics is the level of consistency in the stoichiometric matrix (57, 77, 78), which reflects proper conservation of mass and that no metabolites are incorrectly created or destroyed during simulations. The next metric is a ratio for the quantity of metabolic reactions lacking gene-reaction rules to those possessing associated genes (79), which may indicate an overall confidence in the annotation of the reactions. These features reflect the importance of mass conservation and deliberate gene/reaction annotation which each drive confidence in downstream metabolic predictions, omics data integration, and likelihood for successful downstream experimentation. We found unique challenges within each GENRE which made comparing simulation results across models difficult. Neither iMLTC806cdf nor iHD994 has any detectable gene annotations associated with the reactions it contains. A high degree of stoichiometric matrix inconsistency was detected across icdf834, iHD992, and iCN900; with iHD992 many intracellular metabolites were able to be generated without acquiring necessary precursors from the environment. We also detected structural inconsistencies across several GENREs. For example, those GENREs acquired from the AGORA database possessed several intracellular metabolic products not adequately accounted for biologically (Table S3), as well as mitochondrial compartments despite being bacteria. Additionally, several key C. difficile metabolic pathways were either incomplete or absent from the curated models including multistep Stickland fermentation, membrane-dependent ATP synthase, dipeptide and aminoglycan utilization, and a variety of saccharide fermentation pathways (23). Considering each of these factors, the C. difficile GENREs generated here correct numerous mass and annotation inconsistencies, contain key functional capacities, and phenotypically mimic C. difficile.

10.1128/mSystems.00919-21.8 TABLE S3 Topology summary statistics for C. difficile GENREs from AGORA and those generated here. Download Table S3, XLSX file, 0.01 MB. Copyright © 2021 Jenior et al. 2021 Jenior et al. https://creativecommons.org/licenses/by/4.0/ This content is distributed under the terms of the Creative Commons Attribution 4.0 International license.

### Context-specific modeling to capture virulence-associated metabolism.

Following validation, we sought to utilize each GENRE to predict *in situ* metabolic phenotypes that correspond with expression of known virulence traits in C. difficile. As previously stated, GENREs have provided powerful platforms for the integration of transcriptomic data, creating greater context for the shifts observed between conditions and capturing the potential influence of pathways not obviously connected (80). With this application in mind, we chose to generate context-specific models for both *in vitro* and *in vivo* experimental conditions characterized with transcriptome sequencing (RNA-Seq) analysis utilizing a recently published unsupervised transcriptomic data integration method (18). Briefly, the algorithm calculates the most cost-efficient usage of the metabolic network to achieve growth given the pathway investments indicated by the transcriptomic data. This approach is in line with the concept that natural selection generally selects against wasteful production of cellular machinery (81). The output models contain only those metabolic reactions that are most likely to be active under the given conditions, whose ranges of metabolic reaction activity were subsequently deeply sampled to assess for distinct yet equally optimal combinations of active pathways. Analysis of these distributions affords the ability to make much more fine-scale predictions of metabolic changes that C. difficile undergoes as it activates pathogenicity. The patterns of active pathways also reveal critical elements within context-specific metabolism that could lead to targeted strategies for intentional downregulation of virulence factors through metabolite-focused interventions.

### Phase variation in C. difficile str. R20291 is sensitive to carbohydrate availability.

C. difficile is known to utilize phase variation, a reversible mechanism employed by many bacterial pathogens to generate phenotypic and metabolic heterogeneity to maximize overall fitness of the population. Phase variation has been shown to also influence virulence expression in C. difficile str. R20291 (82). One aspect of this phase variation manifests as a rough- or smooth-edged colony morphology on solid agar; the morphologies can be propagated via subculture and are associated with distinct motility behaviors and altered virulence (83). The colony morphology variants are generated through the phase-variable (on/off) expression of the *cmrRST* genes. Toward understanding this phenotype, we experimentally generated rough and smooth phase variants of C. difficile str. R20291 grown on solid supplemented brain heart infusion (BHIS) rich medium for 48 h and sequenced transcriptomes from both groups. Utilizing these data, we generated context-specific versions of iCdR703 under simulated rich medium conditions and deeply sampled the resultant metabolic flux distributions to assess all possible forms of metabolism given the new constraints.

**FIG 2:**
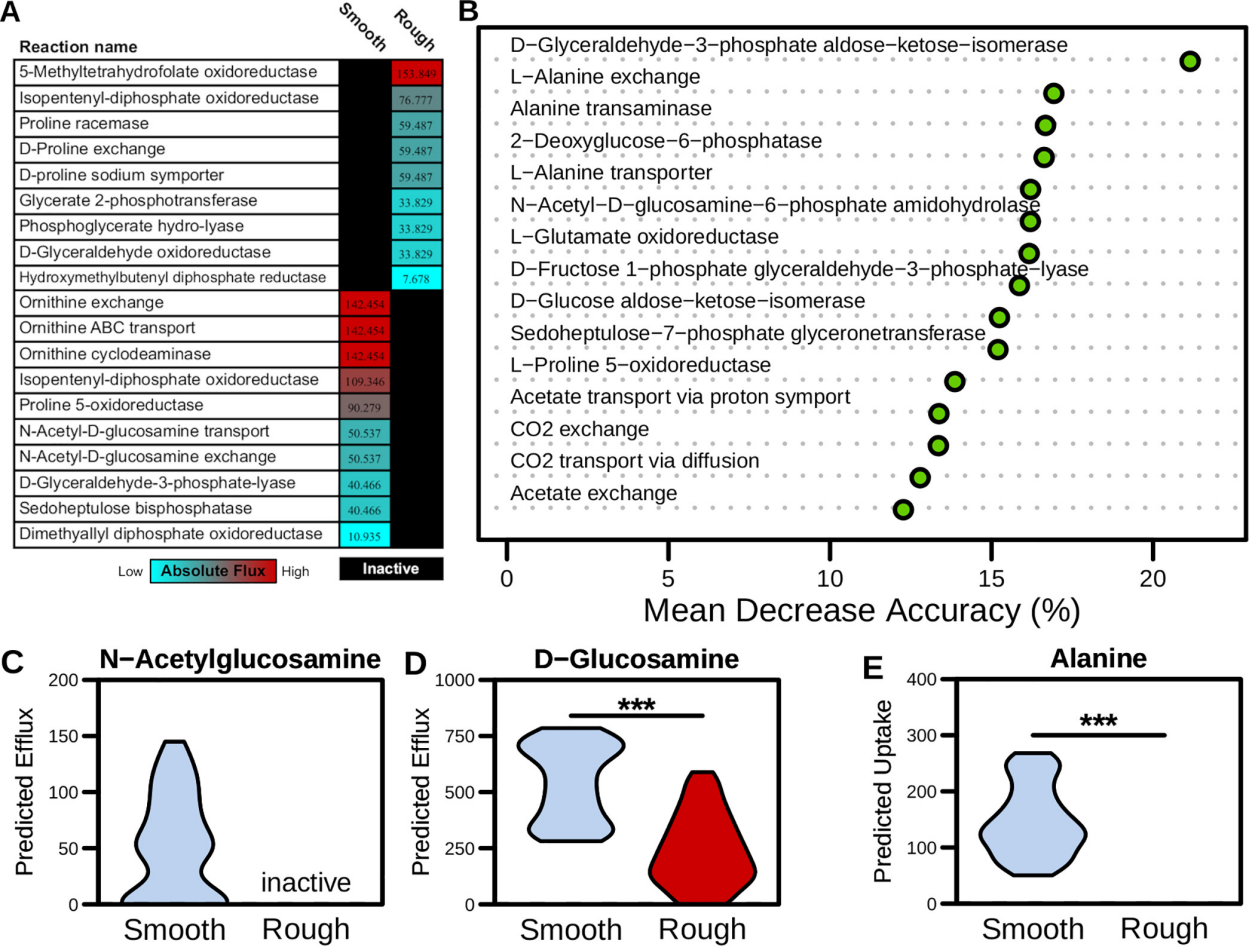
Metabolism significantly varies between phase variants of C. difficile str. R20291. Transcriptomes were collected from rough or smooth colony morphology clones grown on BHIS agar for 48 h and subsequently used to generate context-specific models of C. difficile str. R20291. Subnetworks of metabolism that were predicted to be unused in each context were inactivated for subsequent growth simulations. Context-specific metabolic reaction activity significantly correlated with the associated enzyme transcript abundances (*R* ≥ 0.157, *P* value ≤ 0.023). (A) Metabolic reactions that are uniquely active in each context-specific model and the associated median absolute reaction activities. (B) Utilizing Random Forest supervised machine learning sampled activity for shared nonbiomass metabolic reactions between rough and smooth context-specific models (i.e., core metabolism). Shown is the mean decrease accuracy for the top 15 most differentiating reactions. (C and D) Export exchange reaction flux samples (*n* = 500) between phase variants for *N*-acetylglucosamine and glucosamine (*P* value < 0.001). (E) Import exchange reaction absolute fluxes between phase variants for alanine (*P* value < 0.001). Inactive label denotes reactions pruned during transcriptome contextualization, and all significant differences were determined by Wilcoxon rank sum test.

While it has been previously shown that mutation of *cmr*-family genes does not significantly alter growth rate *in vitro* (83), the contextualized models predicted significantly increased biomass flux generation (reflective of growth rate) with smooth colony-associated metabolism (Fig. S2A). This result fits with experimental findings as the rough-edged phenotype emerges only after long periods of incubation on solid agar when growth rate is measurably lowered (43). We moved on to evaluate structural differences between the context-specific models and identified those metabolic reactions predicted to be active in only the smooth or rough context-specific model. With this analysis we found 19 reactions that were distinctly active between conditions (Fig. 2A). We then calculated median absolute activity for each reaction, which indicated the magnitude at which each reaction contributed to optimal growth in each model. This investigation revealed that proline or ornithine fermentation was present and active in either model (Fig. 2A). C. difficile is capable of easily converting ornithine into proline (52), which is subsequently fermented to 5-aminovalerate for energy. This finding illustrated that proline Stickland fermentation was an integral part of C. difficile metabolism across conditions. The finding that *N*-acetylglucosamine transport was present only within the smooth variant context-specific model was striking as this phase has been previously associated with significantly increased biofilm formation (83), in which *N*-acetyl-d-glucosamine is the primary component (84). Observing the predicted reaction activity, not only was *N*-acetylglucosamine transport present exclusively in the smooth variant context-specific model, but this reaction was extremely active under these conditions (Fig. 2C). Furthermore, efflux of the related metabolite d-glucosamine was also significantly increased in the smooth model (Fig. 2D; *P* value < 0.001). These results supported that the differences in context-specific model structure seen between phase variants likely represented real variation in active metabolism.

10.1128/mSystems.00919-21.2 FIG S2 Predicted core metabolic activity significantly differs between str. 630 phase variants. (A) Sampled biomass objective flux distributions from each context-specific model. Significance determined by Wilcoxon rank sum test (*P* value < 0.001). (B) Analysis limited to nonbiomass reactions shared across context-specific models of iCdR703. NMDS ordination of Bray-Curtis dissimilarities for flux sampling results from core metabolic reactions. Significant difference determined by PERMANOVA. Download FIG S2, PDF file, 0.03 MB. Copyright © 2021 Jenior et al. 2021 Jenior et al. https://creativecommons.org/licenses/by/4.0/ This content is distributed under the terms of the Creative Commons Attribution 4.0 International license.

To then compare metabolic activity effectively between context-specific models, we next focused our analysis on shared nonbiomass associated reactions across context-specific models which we referred to as “core” metabolism within each subsequent analysis. We first employed unsupervised machine learning for flux samples from core reactions using nonmetric multidimensional scaling (NMDS) ordination of Bray-Curtis dissimilarities (Fig. S2B). This analysis revealed significantly different patterns of core metabolic activity between smooth and rough context-specific models (*P* value = 0.001). To further explore the specific differences within active metabolism between phase variants, we utilized a supervised machine learning approach with Random Forest to discriminate between rough and smooth core metabolic activity (Fig. 2B). Several of the metabolic reactions with highest mean decrease accuracies are involved in alanine transport and utilization. Further examination of alanine transport reaction fluxes revealed that import and utilization of alanine were predicted only in the smooth context (Fig. 2E). Alanine has been previously identified as having a strong impact on C. difficile life cycle physiology (85) and has also been shown to be essential for proper biofilm formation in other Gram-positive pathogens (86). Our results indicate that utilization of alanine may also play a role in biofilm formation and phase variation in C. difficile.

**FIG 3:**
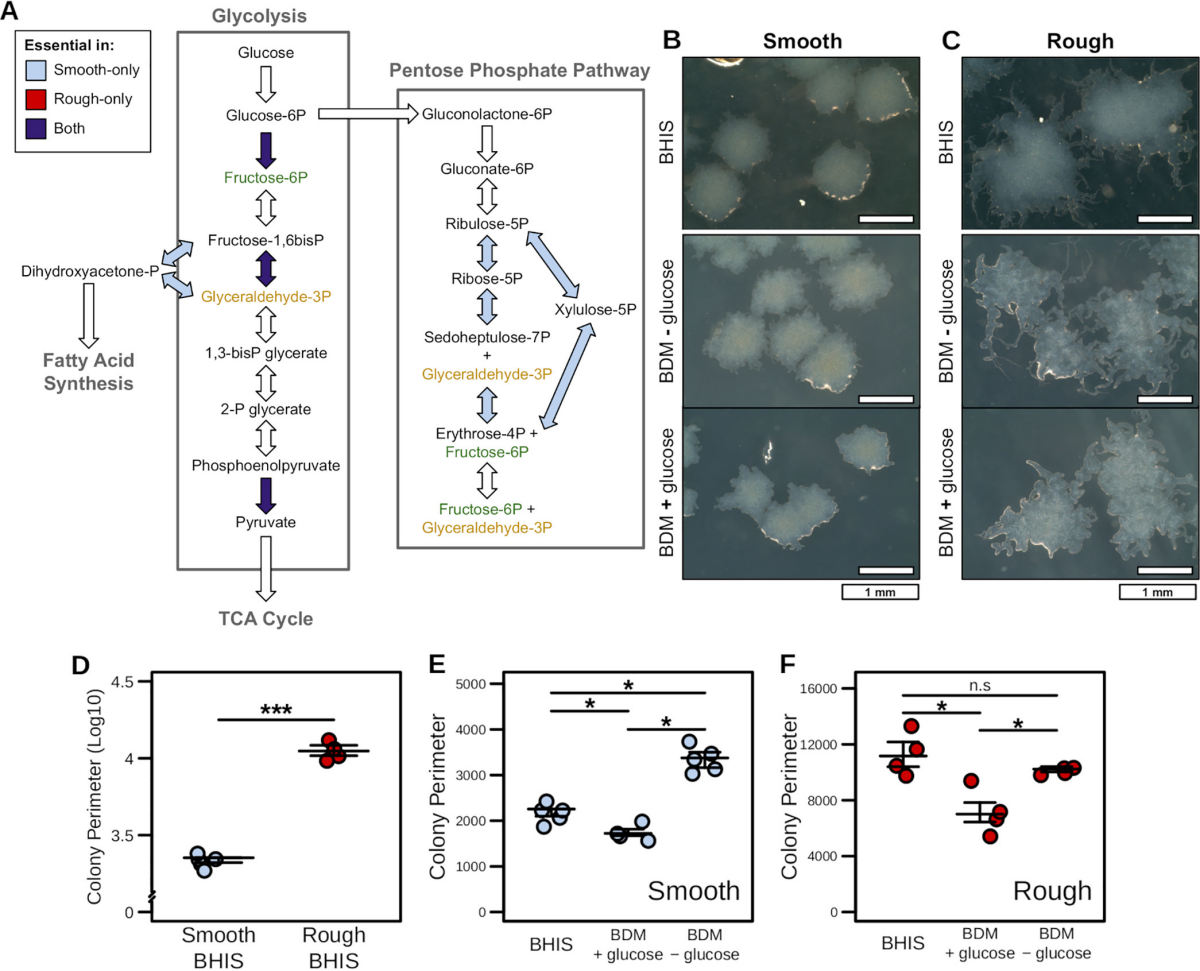
Glucose utilization through the pentose phosphate pathway is essential in the smooth phase variants of str. R20291. (A) Gene and reaction essentiality results for glycolysis and the pentose phosphate pathway across both the rough and smooth phase variant context-specific models. Components were deemed essential if models failed to generate <1% of optimal biomass flux. (B and C) Colony morphologies resulting from smooth and rough variants of C. difficile str. R20291 grown on either BHI or BDM ± glucose (2 mg/ml) after 48 h of growth (phase contrast 20/40, ×4 magnification). Defined medium colonies were then subcultured onto BHI medium for an additional 24 h as indicated. Increased colony perimeter was found to be the defining characteristic of the rough colony morphology. This feature was quantified for multiple colonies under each permutation of colony variant and growth medium (*n* ≥ 4). (D) Colony perimeter for smooth and rough progenitor colony variants grown on BHIS (*P* value < 0.001). (E and F) Smooth (E) or rough (F) colony variant perimeter during subculture onto each of the BDM carbon source medium formulations (*P* values < 0.05). Significant differences determined by Wilcoxon rank sum test with Benjamini-Hochberg correction when necessary.

Both the network topology and metabolic activity-based analyses indicated that a large number of transporters and metabolic reactions were differentially active (Table S4), especially several relating to glycolysis. To more closely investigate the relative importance of these metabolic pathways between phase variants, we performed gene essentiality analysis for both models and cross-referenced the results for metabolic reactions associated with the uptake and utilization of glucose (Fig. 3A). Through this comparison, we found numerous reactions that were essential only in the smooth context-specific model which included multiple steps in the pentose phosphate pathway (involved in nucleotide synthesis and NADPH balance) as well the reactions bridging glycolysis with fatty acid synthesis. Strikingly, no reactions in either pathway were found to be uniquely essential in the rough context-specific model. Although some components of glycolysis were essential in both contexts, including pyruvate kinase, the penultimate step with the bulk of the ATP production was detected at the transcriptional level at nearly identical levels between the rough and smooth isolates (Table S4). These findings together signified that ATP generation from glycolysis was important in both contexts, but the nucleotide precursors and redox potential generated from the pentose phosphate pathway were necessary for the smooth variant-specific metabolism. In line with this observation, the rough context-specific model indeed generated a greater fraction of NADH from Stickland fermentation (Table S4). Based on these data, we hypothesized that this additional dependence on glucose was critical in the smooth variants and without glucose colony morphology would transition toward a more rough phenotype.

10.1128/mSystems.00919-21.9 TABLE S4 Rough versus smooth context-specific analysis of iCdR703 (str. R20291). Download Table S4, XLSX file, 0.03 MB. Copyright © 2021 Jenior et al. 2021 Jenior et al. https://creativecommons.org/licenses/by/4.0/ This content is distributed under the terms of the Creative Commons Attribution 4.0 International license.

To test this hypothesis, we generated colonies of either rough or smooth morphology using C. difficile str. R20291, grown anaerobically for 48 h on BHIS agar (Fig. S3A). We found that the hallmark metric of rough morphology is a significant increase in colony perimeter (Fig. 3D) and used this measurement for determining subsequent shifts between the phenotypes. Both phase variants were subcultured onto BDM agar plates both with and without 2 mg/ml glucose (Fig. 3B and C). Following anaerobic incubation for 48 h, we found that rough variants maintained their morphology across both media, with the rough phenotype even exacerbated on the minimal medium. However, while the smooth variant largely maintained its colony morphology upon subculture onto BDM + glucose, the colonies became significantly rough when glucose was absent (Fig. 3E). The inverse was also true in that the rough colonies maintained their morphology in the absence of glucose but significantly decreased in perimeter on BDM + glucose, appearing more smooth (Fig. 3F). Further subculture of each altered morphology from minimal medium back onto rich BHI medium also appeared to support consistent switching between the respective morphologies (Fig. S3B). Our data supported that the smooth phase variants relied on glucose for more than strictly ATP generation and that the rough morphology is apparent only after extended incubation when C. difficile may be locally activating starvation responses and switching toward alternative energy sources. Additionally, when glucose is available, C. difficile will opt to generate redox potential more efficiently through the pentose phosphate pathway. Furthermore, these results are consistent with the hypothesis that carbohydrate availability impacts phase variation in C. difficile and that environmental stress due to limited nutrients may be a key factor in driving the shift between phases.

10.1128/mSystems.00919-21.3 FIG S3 Additional microscopy of phase variant colony morphologies. (A) C. difficile str. R20291 phase variant progenitor colonies generated on solid BHIS agar following 48 h of growth at 37°C under anaerobic conditions. These colonies were subcultured and utilized for all subsequent defined minimal medium experiments. (B) Subcultured colonies from the indicated conditions in Fig. 3B and C onto BHIS rich agar medium, incubated at 37°C for 48 h anaerobically. Download FIG S3, PDF file, 2.3 MB. Copyright © 2021 Jenior et al. 2021 Jenior et al. https://creativecommons.org/licenses/by/4.0/ This content is distributed under the terms of the Creative Commons Attribution 4.0 International license.

### Utilization of *N*-acetylneuraminic acid and cytidine decreases sporulation in C. difficile str. 630.

While laboratory conditions are highly informative, it is even more critical to examine metabolism for this pathogen during infection as it can more readily lead to novel therapeutic interventions. It has been previously shown that different classes of antibiotics have distinct impacts on the structure of the gut microbiota while inducing similar sensitivity to colonization by C. difficile (87). Along these lines, one published study assessed differential transcriptional activity of C. difficile str. 630 in the gut during infection in a mouse model pretreated with the antibiotic cefoperazone or clindamycin. Crucially, these treatments resulted in highly dissimilar levels of sporulation (another phenotype linked to C. difficile virulence) where cefoperazone had largely undetectable spore CFU and clindamycin had significantly higher levels at the same time point (7). These experiments included paired, untargeted metabolomic analysis of intestinal content to correlate the transcriptional activity of metabolic pathways with changes in the abundance of their respective substrates. Included in the analysis were both mock-infected and C. difficile-colonized groups (both treated by the respective antibiotics) to extract the specific impact of the infection on the gut environment, making this data set extremely valuable.

**FIG 4:**
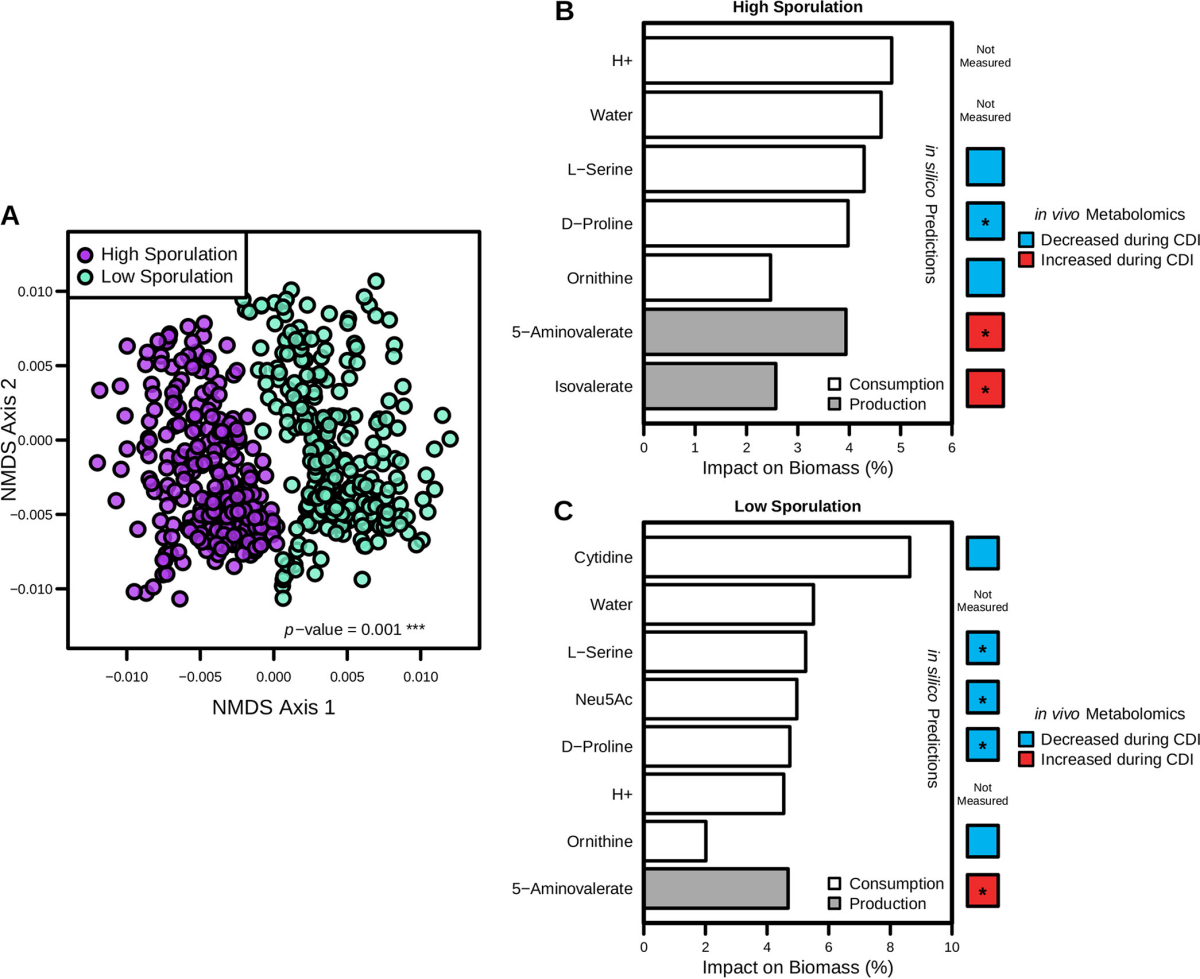
Predicted differences in C. difficile str. 630 carbon source usage correspond with lowered rates of sporulation. Transcriptomic integration and predictions with iCdG709, 18 h after infection with str. 630, across infections with either high or low levels of sporulation were detected in the cecum. Predicted context-specific metabolism significantly correlated with associated transcript values (*R* ≥ 0.188, *P* value ≤ 0.001). (A) NMDS ordination of Bray-Curtis dissimilarities for flux distributions of shared reactions following sampling of context-specific models. Significant difference calculated by PERMANOVA. (B and C) Iterative growth simulations for higher-sporulation context-specific model (B) and in the lower-sporulation context-specific model (C), displaying metabolites with any impact on biomass production when consumption or production capability was restricted to 1.0% of optimal in a given context-specific model. Along the right margin is paired liquid chromatography-mass spectrometry (LC-MS) analysis from cecal content of mice with and without C. difficile str. 630 infection in antibiotic pretreatment groups that resulted in either high or low cecal spore CFU for metabolites highlighted by growth simulation analysis. Each is colored by mean decrease/increase in concentration between mock and infected groups, and asterisks indicate significant differences determined by Wilcoxon rank sum test with Benjamini-Hochberg correction for multiple comparisons (*P* values ≤ 0.05).

We first compared predicted biomass objective flux in the sampled context-specific flux distributions (Fig. S4A), which revealed no significant difference between high- and low-sporulation conditions. However, ordination analysis, performed as described above, indeed revealed significant differences in predicted core metabolic activity (Fig. 4A; *P* value = 0.001). Comparisons of overall context-specific model flux distributions highlighted substantial differences in transporter and metabolic reaction network compositions and associated activities (Table S5). In agreement with these findings, supervised machine learning analysis indicated numerous differences in reactions associated with metabolizing host-derived glycans and nucleotide precursors (Fig. S4B). To focus this assessment on growth substrates that may be differentially impacting the observed levels of sporulation, we assessed each context-specific model by sequentially limiting the ability to import or export each extracellular metabolite to 1% of its optimal rate and measured the impact on overall biomass production (Fig. 4B and C). Paired metabolomic analyses of each metabolite identified this way were then compared within each condition for relative change in concentration following infection, represented as colored squares along the right margin of Fig. 4B and C. Many metabolites had no effect on biomass when their exchange rates were limited and simply rerouted metabolism elsewhere to achieve similar levels of growth, which indicated a high degree of metabolic plasticity remaining in each context-specific model. All metabolites highlighted by this analysis that were measured by the metabolomics screen followed the model-predicted directional change in concentration, supporting the hypothesis that C. difficile itself is responsible for the observed differences (Fig. 4B and C). The peptides proline, ornithine, and serine were found to have an impact on the ability to grow across both context-specific models. Of this subset of amino acids, only proline is an auxotrophy and all are usable by C. difficile in Stickland fermentation. Following the catabolism of proline, its Stickland fermentation by-product 5-aminovalerate was predicted to be an important efflux metabolite under both conditions and had concordant significant increases in concentration following infection in each group (Fig. S4C). Alternatively, isovalerate efflux was found to be critical only in the higher-sporulation context (Fig. 4B). This short-chain fatty acid has been primarily associated with leucine fermentation in C. difficile, supporting an elevated dependence on Stickland fermentation as sporulation increases. Intestinal concentrations of leucine have indeed been shown to significantly decrease following infection by C. difficile *in vivo* (7), supporting its importance during infection. The most distinguishing features were the importance of *N*-acetylneuraminate (Neu5Ac) and cytidine only in the lower-sporulation context-specific model (Fig. 4C). *N*-Acetylneuraminate is a host-derived component of sialic acid that C. difficile readily uses as a carbon source for growth (7), and cytidine is an integral component of RNA synthesis. However, neither had been previously associated with directly influencing virulence factor expression in C. difficile. Furthermore, while *N*-acetylneuraminate significantly decreases during infection in the lower-sporulation context (7), cytidine also appears to decrease under these conditions, implying consumption by C. difficile (Fig. S4D and E).

10.1128/mSystems.00919-21.4 FIG S4 Strain 630 growth simulation and infection metabolomic results. (A) Sampled biomass objective flux distributions from each context-specific model. Significant difference tested using Wilcoxon rank sum test (*P* value = n.s.). (B) Mean decrease accuracy for top 10 most differentiating features/reactions from Random Forest supervised machine learning for shared nonbiomass reactions across context-specific models of iCdG709. (C) Cecal concentrations of 5-aminovalerate in mock- and C. difficile str. 630-infected mice pretreated with streptomycin, 18 h after initial colonization. Significance determined by Wilcoxon rank sum test; *P* values = 0.003 (high spores) and 0.0001 (low spores). Download FIG S4, PDF file, 0.01 MB. Copyright © 2021 Jenior et al. 2021 Jenior et al. https://creativecommons.org/licenses/by/4.0/ This content is distributed under the terms of the Creative Commons Attribution 4.0 International license.

10.1128/mSystems.00919-21.10 TABLE S5 High-sporulation versus low-sporulation context-specific analysis of iCdG709 (str. 630). Download Table S5, XLSX file, 0.03 MB. Copyright © 2021 Jenior et al. 2021 Jenior et al. https://creativecommons.org/licenses/by/4.0/ This content is distributed under the terms of the Creative Commons Attribution 4.0 International license.

**FIG 5:**
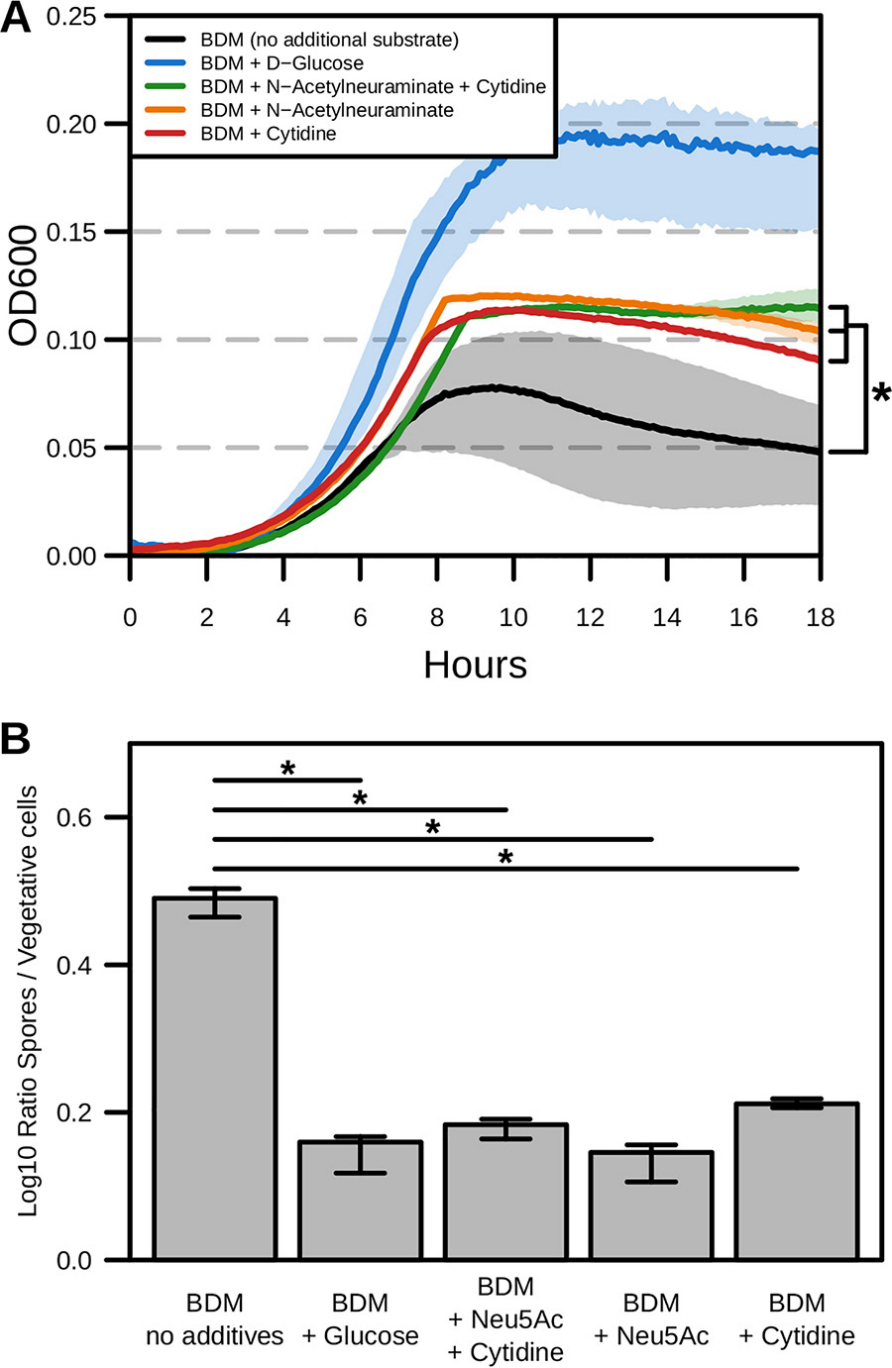
*N*-Acetylneuraminic acid and cytidine drive changes in str. 630 growth and sporulation. (A) Eighteen-hour anaerobic C. difficile str. 630 growth measured at OD_600_ in defined minimal medium (BDM) formulated with the indicated carbon sources (10 mg/ml each; *n* = 4). Significant differences determined using PERMANOVA of Dynamic Time Warping distances (*P* values < 0.05). (B) Median and interquartile range (IQR) for the log-transformed C. difficile str. 630 spore-to-vegetative-cell CFU ratio after 18-h incubation in rich medium or defined minimal medium (BDM) formulated with the indicated carbon sources (*n* = 4). Differential plating performed on BHIS agar ± taurocholate (1.0%). Significance determined by Wilcoxon rank sum test with Benjamini-Hochberg correction (*P* values < 0.05).

We first sought to measure if C. difficile str. 630 could utilize both *N*-acetylneuraminate and cytidine as carbon sources and if together they exerted a combined effect on growth. Both *N*-acetylneuraminate and cytidine were supplemented (10 mg/ml each) in liquid BDM in parallel with liquid BDM with no additional substrate and BDM + d-glucose (10 mg/ml) controls, into which C. difficile str. 630 was inoculated and incubated for 18 h and optical density at 600 nm (OD_600_) was measured every 5 min (Fig. 5A). This assay revealed that C. difficile str. 630 could indeed use *N*-acetylneuraminate and cytidine as carbon sources independently, as each condition allowed for significantly more growth than background BDM alone (*P* values < 0.05). Additionally, there was no discernible effect on growth when the two substrates were added simultaneously. Utilization of the nucleotide precursor cytidine as a carbon source during infection has never been previously described in C. difficile, which further supported the utility of our models as a platform for augmenting discovery.

To then assess the effect of *N*-acetylneuraminate and cytidine on sporulation in C. difficile str. 630, we performed a growth and sporulation assay targeting these substrates using the same defined medium conditions described previously (Fig. 5B). Following 18 h of anaerobic growth, cultures were plated on BHIS agar plates lacking a germination agent to quantify specifically vegetative cell CFU abundance. Remaining liquid cultures were then treated with a final concentration of 50% ethanol (EtOH) for 60 min to eliminate vegetative cells and then plated on BHIS agar with 1% taurocholate added to quantify exclusively spore CFU. The resultant abundances were then converted to an overall spore-to-vegetative-cell ratio to suggest the fraction of the population undergoing sporulation. After overnight incubation, the group that received any combination of *N*-acetylneuraminate or cytidine had significantly decreased levels of sporulation ratios relative to the no-additive control (*P values *< 0.05) but no significant change compared to the glucose-added control (Fig. 5B). Importantly, there were significantly more vegetative cells under all additive conditions relative to BDM alone (Fig. S5; *P* values < 0.05). As was the case in the growth curve results, there was no difference between *N*-acetylneuraminate and cytidine when added alone versus their combined effect. Collectively, these results support that both *N*-acetylneuraminate and cytidine utilization by C. difficile inhibit progression through its life cycle toward spore formation. More broadly, our results support these GENREs as an advantageous discovery platform for novel elements of C. difficile metabolism and physiology.

10.1128/mSystems.00919-21.5 FIG S5 Raw C. difficile str. 630 spore and vegetative cell quantification in defined medium sporulation assay. Median and IQR of C. difficile str. 630 vegetative cells and spore CFU (*n* = 4) after 18-h incubation in rich medium or minimal medium (BDM) formulated with the indicated additional carbon sources (10 mg/ml each). Significant differences calculated with Wilcoxon rank sum test with Benjamini-Hochberg correction (*P* values < 0.05). Download FIG S5, PDF file, 0.06 MB. Copyright © 2021 Jenior et al. 2021 Jenior et al. https://creativecommons.org/licenses/by/4.0/ This content is distributed under the terms of the Creative Commons Attribution 4.0 International license.

## DISCUSSION

The control for much of C. difficile’s physiology and pathogenicity is subject to a coalescence of metabolic signals from both inside and outside the cell. Historically, C. difficile research has suffered from a shortage of molecular tools and high-quality predictive models for highlighting new potential therapies. Over the previous decade, GENREs have become powerful tools for connecting genotype with phenotype and provided platforms for defining novel metabolic targets in biotechnology and improving interpretability of high-dimensional omics data. These factors make GENRE-based analyses extremely promising for directing and accelerating identification of possible therapeutic targets as well as a deeper understanding of the connections between C. difficile virulence and metabolism. Furthermore, as much of bacterial pathogenicity is now being attributed to shifts in metabolism, the analyses described here may provide large benefits to the identification of possible treatment targets in C. difficile and other recalcitrant pathogens (88). In the current study, we develop and validate two highly curated genome-scale metabolic network reconstructions for a well-described laboratory strain (str. 630) in addition to a more recently characterized hypervirulent strain (str. R20291) of C. difficile. Validation results from both models indicated significant agreement with both gene essentiality and carbon utilization screens, indicating a high degree of confidence in subsequent predictions for active metabolism.

We next employed a recently published technique for transcriptome contextualization with data sets from *in vitro* and *in vivo* systems to evaluate potential emergent metabolic drivers of virulence. These combined analyses revealed differential reliance on glycolysis-related metabolism during periods of increased virulence expression. Specifically, in states of elevated biofilm formation by C. difficile str. R20291 we found that glucose is necessary for nucleotide synthesis and redox balance through the pentose phosphate pathway, despite still being utilized for ATP under conditions associated with reduced biofilm. These findings were subsequently supported by direct testing *in vitro* and agreed with recent work which supports that access to glycolysis intermediates actually induces C. difficile biofilm formation (89). Alternatively, during infection with str. 630 we identified patterns of host-derived glycan (*N*-acetylneuraminate) utilization in combination with consumption of the nucleotide precursor cytidine that corresponded with lower levels of sporulation. While not typically considered a carbon source for C. difficile, laboratory testing confirmed that C. difficile can indeed use cytidine for energy and, along with *N*-acetylneuraminate, decreases in sporulation. Intentional control of sporulation is an exciting prospect as spores are considered the transmissive form of C. difficile, so these results may prove valuable for downstream targeted manipulation of C. difficile virulence factor expression. Our results also supported a role for some level of amino acid fermentation across all conditions tested. This phenotype is a hallmark of C. difficile physiology and reinforced the validity of the other predictions. These results indicate a complex relationship with environmental nutrient concentrations and likely competition with the gut microbiota that all inform the regulation of C. difficile virulence expression. Additionally, *in vivo* context-specific gene essentiality also predicted proline racemase to be critical for growth during infection, yet it was previously found to be dispensable in an animal model using a forward genetic screen (90). This result may be attributable to the specific conditions of that infection and may vary across distinct host gut environments, leading to possible implications in personalized medicine and novel approaches to curbing the expression of virulence factors by influencing environmental conditions to favor certain forms of metabolism over others. This study represents the first time that context-specific models of bacterial metabolism have been generated and used to augment discoveries for metabolic control over virulence expression in the laboratory.

While the majority of predictions followed experimental results, several areas of possible expansion and curation are present in both GENREs. First, while the scope of total genes included in iCdG709 and iCdR703 may be more limited than previous network reconstructions, we elected to focus on those gene sets where the greatest amount of evidence and annotation data could be found to maximize confidence in functionality. Consequently, both GENREs consistently underpredict the impact of some metabolite groups, primarily nucleotides and carboxylic acids, which could be due to the absence of annotation or overall knowledge of the relevant cellular machinery. Furthermore, more complex regulatory networks ultimately determine final expression of virulence factors, and these may be needed additions in the future to truly understand the interplay of metabolism and pathogenicity in C. difficile. Despite these potential shortcomings, both iCdG709 and iCdR703 produced highly accurate metabolic predictions for their respective strains as well as novel predictions for metabolism as it relates to C. difficile virulence expression, making both strong candidate platforms for directing future studies of C. difficile metabolic pathways.

Systems-biology approaches have enabled the assessment of fine-scale changes to metabolism of single species within complex environments that may have downstream implications on health and disease. Overall, the combined *in vitro*- and *in vivo*-based results demonstrated that our GENREs are effective platforms for gleaning additional understanding from omics data sets, outside the standard analyses. Both GENREs were able to accurately predict complex metabolic phenotypes when provided context-specific omics data and ultimately underscored the metabolic plasticity of C. difficile. The reciprocal utilization of glycolysis and amino acid fermentation indeed supports regimes of distinct metabolic programming associated with C. difficile pathogenicity. Finding core metabolic properties in C. difficile strains may be key in identifying potential probiotic competitor strains or even molecular inhibitors of metabolic components. The current study is an example of the strength that systems-level analyses have in contributing to more rapid advancements in biological understanding. In the future, the metabolic network reconstructions presented here will be well suited to accelerate research efforts toward the discovery of more targeted therapies. Overall, GENREs have had limited impact to date in real mechanistic understanding of infectious disease, and the current study represents a significant advance in this application.

## MATERIALS AND METHODS

### C. difficile GENRE construction.

We utilized PATRIC reference genomes from Clostridioides difficile str. 630 and Clostridioides difficile str. R20291 as initial reconstruction templates for the automated ModelSEED pipeline (28, 91, 92). The ModelSEED draft network reconstruction was converted utilizing the Mackinac pipeline (https://github.com/mmundy42/mackinac) into a form more compatible with the COBRA toolbox (93). Upon removal of GENRE components lacking genetic evidence (i.e., gap-filled), extensive manual curation was performed in accordance with best practices agreed upon by the community (94). We subsequently performed ensemble gap-filling as previously described, utilizing a stoichiometrically consistent anaerobic, Gram-positive universal reaction collection curated for this purpose and available alongside code associated with this study. Next, we corrected reaction inconsistencies and incorrect physiological properties (e.g., ensured free water diffusion across compartments). Final transport reactions were then validated with TransportDB (95). All formulas are mass and charge balanced at an assumed pH of 7.0 using the ModelSEED database in order to maintain a consistent and supported namespace to augment GENRE interpretability and future curation efforts. We then collected annotation data for all model components (genes, reactions, and metabolites) from SEED (94, 96), KEGG (97), PATRIC, RefSeq (98), EMBL (99), and BiGG (100) databases and integrated them into the annotation field dictionary now supported in the most recent SBML version (101). Complete MEMOTE quality reports for both C. difficile GENREs are also available in the GitHub repository associated with this study, as well as full pipelines for model generation.

### Growth simulations, flux-based analyses, and GENRE quality assessment.

All modeling analyses were carried out using the COBRA toolbox implemented in python (102). The techniques utilized included flux-balance analysis, flux-variability analysis (103), gapsplit flux-sampler (104), and minimal_medium on exhaustive search settings. GENRE quality assessment tools were also developed in python and are fully available in the project GitHub repository. MEMOTE quality reports were generated using the web-based implementation found at https://memote.io/.

### C. difficile str. R20291 *in vitro* growth and microscopy.

C. difficile str. R20291 growth was maintained in an anaerobic environment of 85% N_2_, 5% CO_2_, and 10% H_2_. The strain was grown on BHIS agar (37 g/liter Bacto brain heart infusion, 5 g/liter yeast extract, 1.5% agar) medium at 37°C for 48 h to obtain isolated colonies. Rough and smooth colonies were chosen for propagation on BHI agar to ensure colony morphology maintenance (83). Basal defined medium (BDM) was formulated as previously published (35) with the addition of 1.5% agar for plates and incubated for 48 h at 37°C to generate isolated colonies. Microscopy images were taken on an EVOS XL Core cell imaging system at ×4 magnification. Colony dimensions were determined using ImageJ (https://imagej.nih.gov/ij/).

### C. difficile str. 630 *in vitro* growth and sporulation assay.

C. difficile str. 630 growth was maintained in an anaerobic environment of 85% N_2_, 5% CO_2_, and 10% H_2_. Liquid BDM was formulated as previously described with the indicated combinations of d-glucose (10 mg/ml), *N*-acetylneuraminic acid (10 mg/ml), and cytidine (10 mg/ml). Overnight BHI liquid cultures of C. difficile str. 630 were back-diluted 1:3 in fresh anaerobic BHI and incubated for 1 h at 37°C, at which point 5 μl was inoculated into 1 ml of each medium condition. After 18 h anaerobic incubation at 37°C, serial dilutions in anaerobic phosphate-buffered saline of these cultures were plated on BHIS agar (37 g/liter Bacto brain heart infusion, 5 g/liter yeast extract, 1.5% agar) plates to quantify vegetative cell abundance and then treated with 50% EtOH for 30 min (105), and serial dilutions in anaerobic phosphate-buffered saline were subsequently plated on BHIS agar + 1.0% taurocholate plates to measure spore abundance. Plates were incubated for an additional 24 h at 37°C, at which point CFU were quantified. For anaerobic growth curves, 250 μl of each medium was inoculated with 5 μl of the back-dilution and the OD_600_ was measured every 5 min for 18 h (Tecan Infinite M200 Pro).

### RNA isolation and transcriptome sequencing.

For RNA isolation, rough and smooth isolates were subcultured in BHIS broth (37 g/liter Bacto brain heart infusion, 5 g/liter yeast extract) overnight (16 to 18 h) at 37°C, and then 5 μl of the cultures was spotted on BHIS agar (1.5% agar). After 24 h, the growth was collected and suspended in 1:1 ethanol-acetone for storage at −20°C until subsequent RNA isolation. Cells stored in ethanol-acetone were pelleted by centrifugation and washed in TE (10 mM Tris, 1 mM EDTA, pH 7.6) buffer. Cell pellets were suspended in 1 ml TRIsure reagent. Silica‐glass beads (0.1 mm) were added, and cells were disrupted using bead beating (3,800 rpm) for 1.5 min. Nucleic acids were extracted using chloroform, purified by precipitation in isopropanol followed by washing with cold 70% ethanol, and suspended in nuclease-free water. Samples were submitted to Genewiz, LLC (South Plainfield, NJ, USA), for quality control analysis, DNA removal, library preparation, and sequencing. RNA sample quantification was done using a Qubit 2.0 fluorometer (Life Technologies), and RNA quality was assessed with a 4200 TapeStation (Agilent Technologies). The Ribo Zero rRNA removal kit (Illumina) was used to deplete rRNA from the samples. RNA sequencing library preparation was done using the NEBNext Ultra RNA library prep kit for Illumina (NEB) according to the manufacturer’s protocol. Sequencing libraries were checked using the Qubit 2.0 fluorometer. The libraries were multiplexed for clustering on one lane of the Illumina HiSeq flow cell. The samples were sequenced using a 2 × 150 paired-end configuration on an Illumina HiSeq 2500 instrument. Image analyses and base calling were done using the HiSeq control software. The resulting raw sequence data files (.bcl) were converted to the FASTQ format and demultiplexed with bcl2fastq 2.17 software (Illumina). One mismatch was permitted for index sequence identification. Data were analyzed using CLC Genomics Workbench v. 20 (Qiagen). Reads were mapped to the C. difficile R20291 genome (FN545816.1) using the software’s default scoring penalties for mismatch, deletion, and insertion differences. All samples yielded over 22 million total reads, with over 20 million mapped to the reference (>93% of total reads, and >90% reads in pairs). Transcript reads for each gene were normalized to the total number of reads and gene length (expressed as reads per kilobase of transcript per million mapped reads [RPKM]).

### Genomic and transcriptomic data processing.

Alignment of C. difficile str. 630 and str. R20291 peptide sequences was performed using bidirectional BLASTp. RNA-Seq reads were first quality trimmed with Sickle with a cutoff ≥Q30 (Joshi and Fass, 2011 [106]). Mapping curated reads to the respective C. difficile genome was then performed with Bowtie2 (107). MarkDuplicates then removed optical and PCR duplicates (broadinstitute.github.io/picard/), and mappings were converted to idxstats format using SAMtools (108). Abundances were then normalized to both read and target lengths. Transcriptomic integration and context-specific model generation were performed with RIPTiDe (v3.2.3) using the maxfit_contextualize() function on the default settings (18).

### Statistical methods.

All statistical analysis was performed in R v3.2.0. Nonmetric multidimensional scaling of Bray-Curtis dissimilarity and permutational multivariate analysis of variance (PERMANOVA) analyses were accomplished using the vegan R package (109). Significant differences for single reaction flux distributions, metabolite concentrations, spore CFU, and growth over time were determined by Wilcoxon signed-rank test. Supervised machine learning was accomplished with the implementation of AUC-Random Forest also in R (110). Dissimilarity between C. difficile str. 630 growth curves was determined using Dynamic Time Warping (111).

### Data and code availability.

Genomic and proteomic data for the strains Clostridioides difficile str. 630 (PATRIC ref. 272563.8) and Clostridioides difficile str. R20291 (PATRIC ref. 645463.3) were downloaded from the PATRIC database (91). Transcriptomic data were downloaded in raw FASTQ format from the NCBI Sequence Read Archive (PRJNA415307 and PRJNA354635) and the Gene Expression Omnibus (GSE158225). The GitHub repository for this study, with all programmatic code and GENREs described here, can be found at https://github.com/mjenior/Jenior_CdifficileGENRE_2021.

## References

[1] Lessa FC, Mu Y, Bamberg WM, Beldavs ZG, Dumyati GK, Dunn JR, Farley MM, Holzbauer SM, Meek JI, Phipps EC, Wilson LE, Winston LG, Cohen JA, Limbago BM, Fridkin SK, Gerding DN, McDonald LC, Emerging Infections Program C. difficile Surveillance Team. 2015. Burden of Clostridium difficile infection in the United States. N Engl J Med 372:825–834. doi:10.1056/NEJMoa1408913. 25714160 PMC10966662

[2] Di Bella S, Ascenzi P, Siarakas S, Petrosillo N, di Masi A. 2016. Clostridium difficile toxins A and B: insights into pathogenic properties and extraintestinal effects. Toxins 8:134. doi:10.3390/toxins8050134. PMC4885049 27153087

[3] Thomas C, Stevenson M, Riley TV. 2003. Antibiotics and hospital-acquired Clostridium difficile-associated diarrhoea: a systematic review. J Antimicrob Chemother 51:1339–1350. doi:10.1093/jac/dkg254. 12746372

[4] Antunes LCM, Han J, Ferreira RBR, Lolić P, Borchers CH, Finlay BB. 2011. Effect of antibiotic treatment on the intestinal metabolome. Antimicrob Agents Chemother 55:1494–1503. doi:10.1128/AAC.01664-10. 21282433 PMC3067180

[5] Theriot CM, Koenigsknecht MJ, Carlson PE, Jr, Hatton GE, Nelson AM, Li B, Huffnagle GB, Li JZ, Young VB. 2014. Antibiotic-induced shifts in the mouse gut microbiome and metabolome increase susceptibility to Clostridium difficile infection. Nat Commun 5:3114. doi:10.1038/ncomms4114. 24445449 PMC3950275

[6] Fletcher JR, Erwin S, Lanzas C, Theriot CM. 2018. Shifts in the gut metabolome and Clostridium difficile transcriptome throughout colonization and infection in a mouse model. mSphere 3:e00089-18. doi:10.1128/mSphere.00089-18. 29600278 PMC5874438

[7] Jenior ML, Leslie JL, Young VB, Schloss PD. 2017. *Clostridium difficile* colonizes alternative nutrient niches during infection across distinct murine gut microbiomes. mSystems 2:e00063-17. doi:10.1128/mSystems.00063-17. 28761936 PMC5527303

[8] Jenior ML, Leslie JL, Young VB, Schloss PD. 2018. Clostridium difficile alters the structure and metabolism of distinct cecal microbiomes during initial infection to promote sustained colonization. mSphere 3:e00261-18. doi:10.1128/mSphere.00261-18. 29950381 PMC6021602

[9] Hofmann JD, Otto A, Berges M, Biedendieck R, Michel A-M, Becher D, Jahn D, Neumann-Schaal M. 2018. Metabolic reprogramming of Clostridioides difficile during the stationary phase with the induction of toxin production. Front Microbiol 9:1970. doi:10.3389/fmicb.2018.01970. 30186274 PMC6110889

[10] Dineen SS, McBride SM, Sonenshein AL. 2010. Integration of metabolism and virulence by Clostridium difficile CodY. J Bacteriol 192:5350–5362. doi:10.1128/JB.00341-10. 20709897 PMC2950512

[11] Centers for Disease Control and Prevention. 2019. Antibiotic resistance threats in the United States, 2019. Centers for Disease Control and Prevention, Atlanta, GA.

[12] Merrigan M, Venugopal A, Mallozzi M, Roxas B, Viswanathan VK, Johnson S, Gerding DN, Vedantam G. 2010. Human hypervirulent Clostridium difficile strains exhibit increased sporulation as well as robust toxin production. J Bacteriol 192:4904–4911. doi:10.1128/JB.00445-10. 20675495 PMC2944552

[13] Hao T, Wu D, Zhao L, Wang Q, Wang E, Sun J. 2018. The genome-scale integrated networks in microorganisms. Front Microbiol 9:296. doi:10.3389/fmicb.2018.00296. 29527198 PMC5829631

[14] Pacheco AR, Moel M, Segrè D. 2019. Costless metabolic secretions as drivers of interspecies interactions in microbial ecosystems. Nat Commun 10:103. doi:10.1038/s41467-018-07946-9. 30626871 PMC6327061

[15] Seif Y, Choudhary KS, Hefner Y, Anand A, Yang L, Palsson BO. 2020. Metabolic and genetic basis for auxotrophies in Gram-negative species. Proc Natl Acad Sci USA 117:6264–6273. doi:10.1073/pnas.1910499117. 32132208 PMC7084086

[16] Hadadi N, Pandey V, Chiappino-Pepe A, Morales M, Gallart-Ayala H, Mehl F, Ivanisevic J, Sentchilo V, van der Meer JR. 2020. Mechanistic insights into bacterial metabolic reprogramming from omics-integrated genome-scale models. NPJ Syst Biol Appl 6:4. doi:10.1038/s41540-020-0123-2. 32001719 PMC6946695

[17] Cesur MF, Siraj B, Uddin R, Durmuş S, Çakır T. 2020. Network-based metabolism-centered screening of potential drug targets in Klebsiella pneumoniae at genome scale. Front Cell Infect Microbiol 9:447. doi:10.3389/fcimb.2019.00447. 31993376 PMC6970976

[18] Jenior ML, Moutinho TJ, Jr, Dougherty BV, Papin JA. 2020. Transcriptome-guided parsimonious flux analysis improves predictions with metabolic networks in complex environments. PLoS Comput Biol 16:e1007099. doi:10.1371/journal.pcbi.1007099. 32298268 PMC7188308

[19] Jijakli K, Jensen PA. 2019. Metabolic modeling of Streptococcus mutans reveals complex nutrient requirements of an oral pathogen. mSystems 4:e00529-19. doi:10.1128/mSystems.00529-19. 31662430 PMC6819733

[20] Bosi E, Monk JM, Aziz RK, Fondi M, Nizet V, Palsson BØ. 2016. Comparative genome-scale modelling of Staphylococcus aureus strains identifies strain-specific metabolic capabilities linked to pathogenicity. Proc Natl Acad Sci USA 113:E3801–E3809. doi:10.1073/pnas.1523199113. 27286824 PMC4932939

[21] Monot M, Boursaux-Eude C, Thibonnier M, Vallenet D, Moszer I, Medigue C, Martin-Verstraete I, Dupuy B. 2011. Reannotation of the genome sequence of Clostridium difficile strain 630. J Med Microbiol 60:1193–1199. doi:10.1099/jmm.0.030452-0. 21349987

[22] Stabler RA, He M, Dawson L, Martin M, Valiente E, Corton C, Lawley TD, Sebaihia M, Quail MA, Rose G, Gerding DN, Gibert M, Popoff MR, Parkhill J, Dougan G, Wren BW. 2009. Comparative genome and phenotypic analysis of Clostridium difficile 027 strains provides insight into the evolution of a hypervirulent bacterium. Genome Biol 10:R102. doi:10.1186/gb-2009-10-9-r102. 19781061 PMC2768977

[23] Neumann-Schaal M, Jahn D, Schmidt-Hohagen K. 2019. Metabolism the Difficile way: the key to the success of the pathogen Clostridioides difficile. Front Microbiol 10:219. doi:10.3389/fmicb.2019.00219. 30828322 PMC6384274

[24] Lawley TD, Clare S, Walker AW, Goulding D, Stabler RA, Croucher N, Mastroeni P, Scott P, Raisen C, Mottram L, Fairweather NF, Wren BW, Parkhill J, Dougan G. 2009. Antibiotic treatment of Clostridium difficile carrier mice triggers a supershedder state, spore-mediated transmission, and severe disease in immunocompromised hosts. Infect Immun 77:3661–3669. doi:10.1128/IAI.00558-09. 19564382 PMC2737984

[25] Deakin LJ, Clare S, Fagan RP, Dawson LF, Pickard DJ, West MR, Wren BW, Fairweather NF, Dougan G, Lawley TD. 2012. The Clostridium difficile spo0A gene is a persistence and transmission factor. Infect Immun 80:2704–2711. doi:10.1128/IAI.00147-12. 22615253 PMC3434595

[26] Meylan S, Andrews IW, Collins JJ. 2018. Targeting antibiotic tolerance, pathogen by pathogen. Cell 172:1228–1238. doi:10.1016/j.cell.2018.01.037. 29522744

[27] Dannheim H, Riedel T, Neumann-Schaal M, Bunk B, Schober I, Spröer C, Chibani CM, Gronow S, Liesegang H, Overmann J, Schomburg D. 2017. Manual curation and reannotation of the genomes of Clostridium difficile 630Δerm and C. difficile 630. J Med Microbiol 66:286–293. doi:10.1099/jmm.0.000427. 28357980

[28] Wattam AR, Davis JJ, Assaf R, Boisvert S, Brettin T, Bun C, Conrad N, Dietrich EM, Disz T, Gabbard JL, Gerdes S, Henry CS, Kenyon RW, Machi D, Mao C, Nordberg EK, Olsen GJ, Murphy-Olson DE, Olson R, Overbeek R, Parrello B, Pusch GD, Shukla M, Vonstein V, Warren A, Xia F, Yoo H, Stevens RL. 2017. Improvements to PATRIC, the all-bacterial Bioinformatics Database and Analysis Resource Center. Nucleic Acids Res 45:D535–D542. doi:10.1093/nar/gkw1017. 27899627 PMC5210524

[29] Thiele I, Palsson BØ. 2010. A protocol for generating a high-quality genome-scale metabolic reconstruction. Nat Protoc 5:93–121. doi:10.1038/nprot.2009.203. 20057383 PMC3125167

[30] Devoid S, Overbeek R, DeJongh M, Vonstein V, Best AA, Henry C. 2013. Automated genome annotation and metabolic model reconstruction in the SEED and Model SEED. Methods Mol Biol 985:17–45. doi:10.1007/978-1-62703-299-5_2. 23417797

[31] Girinathan BP, Braun S, Sirigireddy AR, Espinola-Lopez J, Govind R. 2016. Correction: importance of glutamate dehydrogenase (GDH) in Clostridium difficile colonization in vivo. PLoS One 11:e0165579. doi:10.1371/journal.pone.0165579. 27467167 PMC4965041

[32] Bakker D, Buckley AM, de Jong A, van Winden VJC, Verhoeks JPA, Kuipers OP, Douce GR, Kuijper EJ, Smits WK, Corver J. 2014. The HtrA-like protease CD3284 modulates virulence of Clostridium difficile. Infect Immun 82:4222–4232. doi:10.1128/IAI.02336-14. 25047848 PMC4187886

[33] Mendoza SN, Olivier BG, Molenaar D, Teusink B. 2019. A systematic assessment of current genome-scale metabolic reconstruction tools. Genome Biol 20:158. doi:10.1186/s13059-019-1769-1. 31391098 PMC6685185

[34] Janoir C, Denève C, Bouttier S, Barbut F, Hoys S, Caleechum L, Chapetón-Montes D, Pereira FC, Henriques AO, Collignon A, Monot M, Dupuy B. 2013. Adaptive strategies and pathogenesis of Clostridium difficile from in vivo transcriptomics. Infect Immun 81:3757–3769. doi:10.1128/IAI.00515-13. 23897605 PMC3811758

[35] Karasawa T, Ikoma S, Yamakawa K, Nakamura S. 1995. A defined growth medium for Clostridium difficile. Microbiology (Reading) 141 (Pt 2):371–375. doi:10.1099/13500872-141-2-371. 7704267

[36] Karlsson S, Burman LG, Åkerlund T. 1999. Suppression of toxin production in Clostridium difficile VPI 10463 by amino acids. Microbiology (Reading) 145 (Pt 7):1683–1693. doi:10.1099/13500872-145-7-1683. 10439407

[37] Haslam SC, Ketley JM, Mitchell TJ, Stephen J, Burdon DW, Candy DC. 1986. Growth of Clostridium difficile and production of toxins A and B in complex and defined media. J Med Microbiol 21:293–297. doi:10.1099/00222615-21-4-293. 3088279

[38] Stiemsma LT, Turvey SE, Finlay BB. 2014. An antibiotic-altered microbiota provides fuel for the enteric foe. Cell Res 24:5–6. doi:10.1038/cr.2013.142. 24165893 PMC3879700

[39] Olson ME, King JM, Yahr TL, Horswill AR. 2013. Sialic acid catabolism in Staphylococcus aureus. J Bacteriol 195:1779–1788. doi:10.1128/JB.02294-12. 23396916 PMC3624546

[40] Engevik MA, Yacyshyn MB, Engevik KA, Wang J, Darien B, Hassett DJ, Yacyshyn BR, Worrell RT. 2015. Human Clostridium difficile infection: altered mucus production and composition. Am J Physiol Gastrointest Liver Physiol 308:G510–G524. doi:10.1152/ajpgi.00091.2014. 25552581 PMC4422372

[41] Nakamura S, Nakashio S, Yamakawa K, Tanabe N, Nishida S. 1982. Carbohydrate fermentation by Clostridium difficile. Microbiol Immunol 26:107–111. doi:10.1111/j.1348-0421.1982.tb00159.x. 6806571

[42] Kim J, Darley D, Buckel W. 2005. 2-Hydroxyisocaproyl-CoA dehydratase and its activator from Clostridium difficile. FEBS J 272:550–561. doi:10.1111/j.1742-4658.2004.04498.x. 15654892

[43] Neumann-Schaal M, Hofmann JD, Will SE, Schomburg D. 2015. Time-resolved amino acid uptake of Clostridium difficile 630Δerm and concomitant fermentation product and toxin formation. BMC Microbiol 15:281. doi:10.1186/s12866-015-0614-2. 26680234 PMC4683695

[44] Selmer T, Andrei PI. 2001. p-Hydroxyphenylacetate decarboxylase from Clostridium difficile. A novel glycyl radical enzyme catalysing the formation of p-cresol. Eur J Biochem 268:1363–1372. doi:10.1046/j.1432-1327.2001.02001.x. 11231288

[45] Bouillaut L, Self WT, Sonenshein AL. 2013. Proline-dependent regulation of Clostridium difficile Stickland metabolism. J Bacteriol 195:844–854. doi:10.1128/JB.01492-12. 23222730 PMC3562115

[46] Jackson S, Calos M, Myers A, Self WT. 2006. Analysis of proline reduction in the nosocomial pathogen Clostridium difficile. J Bacteriol 188:8487–8495. doi:10.1128/JB.01370-06. 17041035 PMC1698225

[47] Kim J, Darley D, Selmer T, Buckel W. 2006. Characterization of (R)-2-hydroxyisocaproate dehydrogenase and a family III coenzyme a transferase involved in reduction of L-leucine to isocaproate by Clostridium difficile. Appl Environ Microbiol 72:6062–6069. doi:10.1128/AEM.00772-06. 16957230 PMC1563608

[48] Britz ML, Wilkinson RG. 1982. Leucine dissimilation to isovaleric and isocaproic acids by cell suspensions of amino acid fermenting anaerobes: the Stickland reaction revisited. Can J Microbiol 28:291–300. doi:10.1139/m82-043. 6805929

[49] Kim J, Hetzel M, Boiangiu CD, Buckel W. 2004. Dehydration of (R)-2-hydroxyacyl-CoA to enoyl-CoA in the fermentation of α-amino acids by anaerobic bacteria. FEMS Microbiol Rev 28:455–468. doi:10.1016/j.femsre.2004.03.001. 15374661

[50] Yu L, Blaser M, Andrei PI, Pierik AJ, Selmer T. 2006. 4-Hydroxyphenylacetate decarboxylases: properties of a novel subclass of glycyl radical enzyme systems. Biochemistry 45:9584–9592. doi:10.1021/bi060840b. 16878993

[51] de Vladar HP. 2012. Amino acid fermentation at the origin of the genetic code. Biol Direct 7:6. doi:10.1186/1745-6150-7-6. 22325238 PMC3376031

[52] Fonknechten N, Perret A, Perchat N, Tricot S, Lechaplais C, Vallenet D, Vergne C, Zaparucha A, Le Paslier D, Weissenbach J, Salanoubat M. 2009. A conserved gene cluster rules anaerobic oxidative degradation of L-ornithine. J Bacteriol 191:3162–3167. doi:10.1128/JB.01777-08. 19251850 PMC2681807

[53] Esquivel-Elizondo S, Ilhan ZE, Garcia-Peña EI, Krajmalnik-Brown R. 2017. Insights into butyrate production in a controlled fermentation system via gene predictions. mSystems 2:e00051-17. doi:10.1128/mSystems.00051-17. 28761933 PMC5516221

[54] Ferreyra JA, Wu KJ, Hryckowian AJ, Bouley DM, Weimer BC, Sonnenburg JL. 2014. Gut microbiota-produced succinate promotes C. difficile infection after antibiotic treatment or motility disturbance. Cell Host Microbe 16:770–777. doi:10.1016/j.chom.2014.11.003. 25498344 PMC4859344

[55] Louis P, Flint HJ. 2017. Formation of propionate and butyrate by the human colonic microbiota. Environ Microbiol 19:29–41. doi:10.1111/1462-2920.13589. 27928878

[56] Köpke M, Straub M, Dürre P. 2013. Clostridium difficile is an autotrophic bacterial pathogen. PLoS One 8:e62157. doi:10.1371/journal.pone.0062157. 23626782 PMC3633928

[57] Fritzemeier CJ, Hartleb D, Szappanos B, Papp B, Lercher MJ. 2017. Erroneous energy-generating cycles in published genome scale metabolic networks: identification and removal. PLoS Comput Biol 13:e1005494. doi:10.1371/journal.pcbi.1005494. 28419089 PMC5413070

[58] Nawrocki KL, Wetzel D, Jones JB, Woods EC, McBride SM. 2018. Ethanolamine is a valuable nutrient source that impacts Clostridium difficile pathogenesis. Environ Microbiol 20:1419–1435. doi:10.1111/1462-2920.14048. 29349925 PMC5903940

[59] Senger RS, Papoutsakis ET. 2008. Genome-scale model for Clostridium acetobutylicum: Part I. Metabolic network resolution and analysis. Biotechnol Bioeng 101:1036–1052. doi:10.1002/bit.22010. 18767192 PMC2760220

[60] Lachance J-C, Lloyd CJ, Monk JM, Yang L, Sastry AV, Seif Y, Palsson BO, Rodrigue S, Feist AM, King ZA, Jacques P-É. 2019. BOFdat: generating biomass objective functions for genome-scale metabolic models from experimental data. PLoS Comput Biol 15:e1006971. doi:10.1371/journal.pcbi.1006971. 31009451 PMC6497307

[61] Biggs MB, Papin JA. 2017. Managing uncertainty in metabolic network structure and improving predictions using EnsembleFBA. PLoS Comput Biol 13:e1005413. doi:10.1371/journal.pcbi.1005413. 28263984 PMC5358886

[62] Medlock GL, Moutinho TJ, Papin JA. 2020. Medusa: software to build and analyze ensembles of genome-scale metabolic network reconstructions. PLoS Comput Biol 16:e1007847. doi:10.1371/journal.pcbi.1007847. 32348298 PMC7213742

[63] Satish Kumar V, Dasika MS, Maranas CD. 2007. Optimization based automated curation of metabolic reconstructions. BMC Bioinformatics 8:212. doi:10.1186/1471-2105-8-212. 17584497 PMC1933441

[64] Atlas RM. 2010. Handbook of microbiological media, 4th ed. CRC Press, Boca Raton, FL.

[65] Blazier AS, Papin JA. 2019. Reconciling high-throughput gene essentiality data with metabolic network reconstructions. PLoS Comput Biol 15:e1006507. doi:10.1371/journal.pcbi.1006507. 30973869 PMC6478342

[66] O’Brien EJ, Monk JM, Palsson BO. 2015. Using genome-scale models to predict biological capabilities. Cell 161:971–987. doi:10.1016/j.cell.2015.05.019. 26000478 PMC4451052

[67] Dembek M, Barquist L, Boinett CJ, Cain AK, Mayho M, Lawley TD, Fairweather NF, Fagan RP. 2015. High-throughput analysis of gene essentiality and sporulation in Clostridium difficile. mBio 6:e02383-14. doi:10.1128/mBio.02383-14. 25714712 PMC4358009

[68] Dubois T, Dancer-Thibonnier M, Monot M, Hamiot A, Bouillaut L, Soutourina O, Martin-Verstraete I, Dupuy B. 2016. Control of Clostridium difficile physiopathology in response to cysteine availability. Infect Immun 84:2389–2405. doi:10.1128/IAI.00121-16. 27297391 PMC4962627

[69] Scaria J, Chen J-W, Useh N, He H, McDonough SP, Mao C, Sobral B, Chang Y-F. 2014. Comparative nutritional and chemical phenome of Clostridium difficile isolates determined using phenotype microarrays. Int J Infect Dis 27:20–25. doi:10.1016/j.ijid.2014.06.018. 25130165 PMC4197074

[70] Battaglioli EJ, Hale VL, Chen J, Jeraldo P, Ruiz-Mojica C, Schmidt BA, Rekdal VM, Till LM, Huq L, Smits SA, Moor WJ, Jones-Hall Y, Smyrk T, Khanna S, Pardi DS, Grover M, Patel R, Chia N, Nelson H, Sonnenburg JL, Farrugia G, Kashyap PC. 2018. Clostridioides difficile uses amino acids associated with gut microbial dysbiosis in a subset of patients with diarrhea. Sci Transl Med 10:eaam7019. doi:10.1126/scitranslmed.aam7019. 30355801 PMC6537101

[71] Larocque M, Chénard T, Najmanovich R. 2014. A curated C. difficile strain 630 metabolic network: prediction of essential targets and inhibitors. BMC Syst Biol 8:117. doi:10.1186/s12918-014-0117-z. 25315994 PMC4207893

[72] Kashaf SS, Angione C, Lió P. 2017. Making life difficult for Clostridium difficile: augmenting the pathogen’s metabolic model with transcriptomic and codon usage data for better therapeutic target characterization. BMC Syst Biol 11:25. doi:10.1186/s12918-017-0395-3. 28209199 PMC5314682

[73] Norsigian CJ, Danhof HA, Brand CK, Oezguen N, Midani FS, Palsson BO, Savidge TC, Britton RA, Spinler JK, Monk JM. 2020. Systems biology analysis of the Clostridioides difficile core-genome contextualizes microenvironmental evolutionary pressures leading to genotypic and phenotypic divergence. NPJ Syst Biol Appl 6:31. doi:10.1038/s41540-020-00151-9. 33082337 PMC7576604

[74] Dannheim H, Will SE, Schomburg D, Neumann-Schaal M. 2017. Clostridioides difficile630Δerm in silico and in vivo - quantitative growth and extensive polysaccharide secretion. FEBS Open Bio 7:602–615. doi:10.1002/2211-5463.12208. PMC5377389 28396843

[75] Hussain HA, Roberts AP, Mullany P. 2005. Generation of an erythromycin-sensitive derivative of Clostridium difficile strain 630 (630Δerm) and demonstration that the conjugative transposon Tn916ΔE enters the genome of this strain at multiple sites. J Med Microbiol 54:137–141. doi:10.1099/jmm.0.45790-0. 15673506

[76] Magnúsdóttir S, Heinken A, Kutt L, Ravcheev DA, Bauer E, Noronha A, Greenhalgh K, Jäger C, Baginska J, Wilmes P, Fleming RMT, Thiele I. 2017. Generation of genome-scale metabolic reconstructions for 773 members of the human gut microbiota. Nat Biotechnol 35:81–89. doi:10.1038/nbt.3703. 27893703

[77] Gevorgyan A, Poolman MG, Fell DA. 2008. Detection of stoichiometric inconsistencies in biomolecular models. Bioinformatics 24:2245–2251. doi:10.1093/bioinformatics/btn425. 18697772

[78] Schellenberger J, Lewis NE, Palsson BØ. 2011. Elimination of thermodynamically infeasible loops in steady-state metabolic models. Biophys J 100:544–553. doi:10.1016/j.bpj.2010.12.3707. 21281568 PMC3030201

[79] Ravikrishnan A, Raman K. 2015. Critical assessment of genome-scale metabolic networks: the need for a unified standard. Brief Bioinform 16:1057–1068. doi:10.1093/bib/bbv003. 25725218

[80] Blazier AS, Papin JA. 2012. Integration of expression data in genome-scale metabolic network reconstructions. Front Physiol 3:299. doi:10.3389/fphys.2012.00299. 22934050 PMC3429070

[81] Lewis NE, Hixson KK, Conrad TM, Lerman JA, Charusanti P, Polpitiya AD, Adkins JN, Schramm G, Purvine SO, Lopez-Ferrer D, Weitz KK, Eils R, König R, Smith RD, Palsson BØ. 2010. Omic data from evolved E. coli are consistent with computed optimal growth from genome-scale models. Mol Syst Biol 6:390. doi:10.1038/msb.2010.47. 20664636 PMC2925526

[82] Anjuwon-Foster BR, Tamayo R. 2017. A genetic switch controls the production of flagella and toxins in Clostridium difficile. PLoS Genet 13:e1006701. doi:10.1371/journal.pgen.1006701. 28346491 PMC5386303

[83] Garrett EM, Sekulovic O, Wetzel D, Jones JB, Edwards AN, Vargas-Cuebas G, McBride SM, Tamayo R. 2019. Phase variation of a signal transduction system controls Clostridioides difficile colony morphology, motility, and virulence. PLoS Biol 17:e3000379. doi:10.1371/journal.pbio.3000379. 31658249 PMC6837544

[84] Dubois T, Tremblay YDN, Hamiot A, Martin-Verstraete I, Deschamps J, Monot M, Briandet R, Dupuy B. 2019. A microbiota-generated bile salt induces biofilm formation in Clostridium difficile. NPJ Biofilms Microbiomes 5:14. doi:10.1038/s41522-019-0087-4. 31098293 PMC6509328

[85] Shrestha R, Lockless SW, Sorg JA. 2017. A Clostridium difficile alanine racemase affects spore germination and accommodates serine as a substrate. J Biol Chem 292:10735–10742. doi:10.1074/jbc.M117.791749. 28487371 PMC5481577

[86] Qiu W, Zheng X, Wei Y, Zhou X, Zhang K, Wang S, Cheng L, Li Y, Ren B, Xu X, Li Y, Li M. 2016. d-Alanine metabolism is essential for growth and biofilm formation of Streptococcus mutans. Mol Oral Microbiol 31:435–444. doi:10.1111/omi.12146. 26526529

[87] Schubert AM, Sinani H, Schloss PD. 2015. Antibiotic-induced alterations of the murine gut microbiota and subsequent effects on colonization resistance against Clostridium difficile. mBio 6:e00974-15. doi:10.1128/mBio.00974-15. 26173701 PMC4502226

[88] Raškevičius V, Mikalayeva V, Antanavičiūtė I, Ceslevičienė I, Skeberdis VA, Kairys V, Bordel S. 2018. Genome scale metabolic models as tools for drug design and personalized medicine. PLoS One 13:e0190636. doi:10.1371/journal.pone.0190636. 29304175 PMC5755790

[89] Tremblay YDN, Durand BAR, Hamiot A, Martin-Verstraete I, Oberkampf M, Monot M, Dupuy B. 2021. Metabolic adaption to extracellular pyruvate triggers biofilm formation in Clostridioides difficile. ISME J doi:10.1038/s41396-021-01042-5. PMC8630010 34155333

[90] Wu X, Hurdle JG. 2014. The Clostridium difficile proline racemase is not essential for early logarithmic growth and infection. Can J Microbiol 60:251–254. doi:10.1139/cjm-2013-0903. 24693984 PMC4076780

[91] Wattam AR, Abraham D, Dalay O, Disz TL, Driscoll T, Gabbard JL, Gillespie JJ, Gough R, Hix D, Kenyon R, Machi D, Mao C, Nordberg EK, Olson R, Overbeek R, Pusch GD, Shukla M, Schulman J, Stevens RL, Sullivan DE, Vonstein V, Warren A, Will R, Wilson MJC, Yoo HS, Zhang C, Zhang Y, Sobral BW. 2014. PATRIC, the bacterial bioinformatics database and analysis resource. Nucleic Acids Res 42:D581–D591. doi:10.1093/nar/gkt1099. 24225323 PMC3965095

[92] Faria JP, Rocha M, Rocha I, Henry CS. 2018. Methods for automated genome-scale metabolic model reconstruction. Biochem Soc Trans 46:931–936. doi:10.1042/BST20170246. 30065105

[93] Heirendt L, Arreckx S, Pfau T, Mendoza SN, Richelle A, Heinken A, Haraldsdóttir HS, Wachowiak J, Keating SM, Vlasov V, Magnusdóttir S, Ng CY, Preciat G, Žagare A, Chan SHJ, Aurich MK, Clancy CM, Modamio J, Sauls JT, Noronha A, Bordbar A, Cousins B, El Assal DC, Valcarcel LV, Apaolaza I, Ghaderi S, Ahookhosh M, Ben Guebila M, Kostromins A, Sompairac N, Le HM, Ma D, Sun Y, Wang L, Yurkovich JT, Oliveira MAP, Vuong PT, El Assal LP, Kuperstein I, Zinovyev A, Hinton HS, Bryant WA, Aragón Artacho FJ, Planes FJ, Stalidzans E, Maass A, Vempala S, Hucka M, Saunders MA, Maranas CD, Lewis NE, Sauter T, Palsson BØ, Thiele I, Fleming RMT. 2019. Creation and analysis of biochemical constraint-based models using the COBRA Toolbox v.3.0. Nat Protoc 14:639–702. doi:10.1038/s41596-018-0098-2. 30787451 PMC6635304

[94] Gu C, Kim GB, Kim WJ, Kim HU, Lee SY. 2019. Current status and applications of genome-scale metabolic models. Genome Biol 20:121. doi:10.1186/s13059-019-1730-3. 31196170 PMC6567666

[95] Ren Q, Chen K, Paulsen IT. 2007. TransportDB: a comprehensive database resource for cytoplasmic membrane transport systems and outer membrane channels. Nucleic Acids Res 35:D274–D279. doi:10.1093/nar/gkl925. 17135193 PMC1747178

[96] Seaver SMD, Liu F, Zhang Q, Jeffryes J, Faria JP, Edirisinghe JN, Mundy M, Chia N, Noor E, Beber ME, Best AA, DeJongh M, Kimbrel JA, D’haeseleer P, Pearson E, Canon S, Wood-Charlson EM, Cottingham RW, Arkin AP, Henry CS. 2021. The ModelSEED Biochemistry Database for the integration of metabolic annotations and the reconstruction, comparison, and analysis of metabolic models for plants, fungi, and microbes. Nucleic Acids Res 49 (D1):D575–D588. doi:10.1093/nar/gkaa746. 32986834 PMC7778927

[97] Kanehisa M, Goto S. 2000. KEGG: Kyoto Encyclopedia of Genes and Genomes. Nucleic Acids Res 28:27–30. doi:10.1093/nar/28.1.27. 10592173 PMC102409

[98] Pruitt KD, Tatusova T, Maglott DR. 2007. NCBI reference sequences (RefSeq): a curated non-redundant sequence database of genomes, transcripts and proteins. Nucleic Acids Res 35:D61–D65. doi:10.1093/nar/gkl842. 17130148 PMC1716718

[99] Baker W, van den Broek A, Camon E, Hingamp P, Sterk P, Stoesser G, Tuli MA. 2000. The EMBL nucleotide sequence database. Nucleic Acids Res 28:19–23. doi:10.1093/nar/28.1.19. 10592171 PMC102461

[100] Norsigian CJ, Pusarla N, McConn JL, Yurkovich JT, Dräger A, Palsson BO, King Z. 2020. BiGG Models 2020: multi-strain genome-scale models and expansion across the phylogenetic tree. Nucleic Acids Res 48:D402–D406. doi:10.1093/nar/gkz1054. 31696234 PMC7145653

[101] Hucka M, Bergmann FT, Chaouiya C, Dräger A, Hoops S, Keating SM, König M, Novère NL, Myers CJ, Olivier BG, Sahle S, Schaff JC, Sheriff R, Smith LP, Waltemath D, Wilkinson DJ, Zhang F. 2019. The Systems Biology Markup Language (SBML): language specification for level 3 version 2 core release 2. J Integr Bioinform 16:20190021. doi:10.1515/jib-2019-0021. PMC6798823 31219795

[102] Ebrahim A, Lerman JA, Palsson BO, Hyduke DR. 2013. COBRApy: COnstraints-Based Reconstruction and Analysis for Python. BMC Syst Biol 7:74. doi:10.1186/1752-0509-7-74. 23927696 PMC3751080

[103] Gudmundsson S, Thiele I. 2010. Computationally efficient flux variability analysis. BMC Bioinformatics 11:489. doi:10.1186/1471-2105-11-489. 20920235 PMC2963619

[104] Keaty TC, Jensen PA. 2020. gapsplit: efficient random sampling for non-convex constraint-based models. Bioinformatics 36:2623–2625. doi:10.1093/bioinformatics/btz971. 31913465 PMC7178416

[105] Borriello SP, Honour P. 1981. Simplified procedure for the routine isolation of Clostridium difficile from faeces. J Clin Pathol 34:1124–1127. doi:10.1136/jcp.34.10.1124. 7031097 PMC494377

[106] Joshi NA, Fass JN. 2011. Sickle: a sliding-window, adaptive, quality-based trimming tool for FastQ files (version 1.33). https://github.com/najoshi/sickle.

[107] Langmead B, Salzberg SL. 2012. Fast gapped-read alignment with Bowtie 2. Nat Methods 9:357–359. doi:10.1038/nmeth.1923. 22388286 PMC3322381

[108] Li H, Handsaker B, Wysoker A, Fennell T, Ruan J, Homer N, Marth G, Abecasis G, Durbin R, 1000 Genome Project Data Processing Subgroup. 2009. The Sequence Alignment/Map format and SAMtools. Bioinformatics 25:2078–2079. doi:10.1093/bioinformatics/btp352. 19505943 PMC2723002

[109] Dixon P. 2003. VEGAN, a package of R functions for community ecology. J Veg Sci 14:927–930. doi:10.1111/j.1654-1103.2003.tb02228.x.

[110] Janitza S, Strobl C, Boulesteix A-L. 2013. An AUC-based permutation variable importance measure for random forests. BMC Bioinformatics 14:119. doi:10.1186/1471-2105-14-119. 23560875 PMC3626572

[111] Giorgino T. 2009. Computing and visualizing dynamic time warping alignments in R: the dtw package. J Stat Softw 31 (7):1–24. doi:10.18637/jss.v031.i07.

