# Supplementary material for: Novel Drivers of Virulence in Clostridioides difficile Identified via Context-Specific Metabolic Network Analysis
Source: mSystems. 2021 Oct 5;6(5):e00919-21. doi: 10.1128/mSystems.00919-21 (PMC8547418; doi:10.1128/mSystems.00919-21)

A

iCdR703 (str. R20291)

Dembek et al. (2015)

Prediction	Essential	Non-essential
Essential	45 (78.95%)	12 (21.05%)
Non-essential	64 (9.95%)	579 (90.05%)

Accuracy: 89.1%

iCdG709 (str. 630)

str. R20291 homologs

Prediction	Essential	Non-essential
Essential	44 (78.57%)	12 (21.43%)
Non-essential	64 (10.14%)	567 (89.86%)

Accuracy: 88.9%

B

iCdR703 (str. R20291)

Scaria et al. (2014)

Prediction	Enhances	No Enhancement
Enhances	78 (95.12%)	4 (4.88%)
No Enhancement	32 (29.09%)	1 (3.03%)

Positive predictive value: 95.1%

iCdG709 (str. 630)

Scaria et al. (2014)

Prediction	Enhances	No Enhancement
Enhances	76 (92.68%)	6 (7.32%)
No Enhancement	32 (29.63%)	1 (3.03%)

Positive predictive value: 92.3%

C

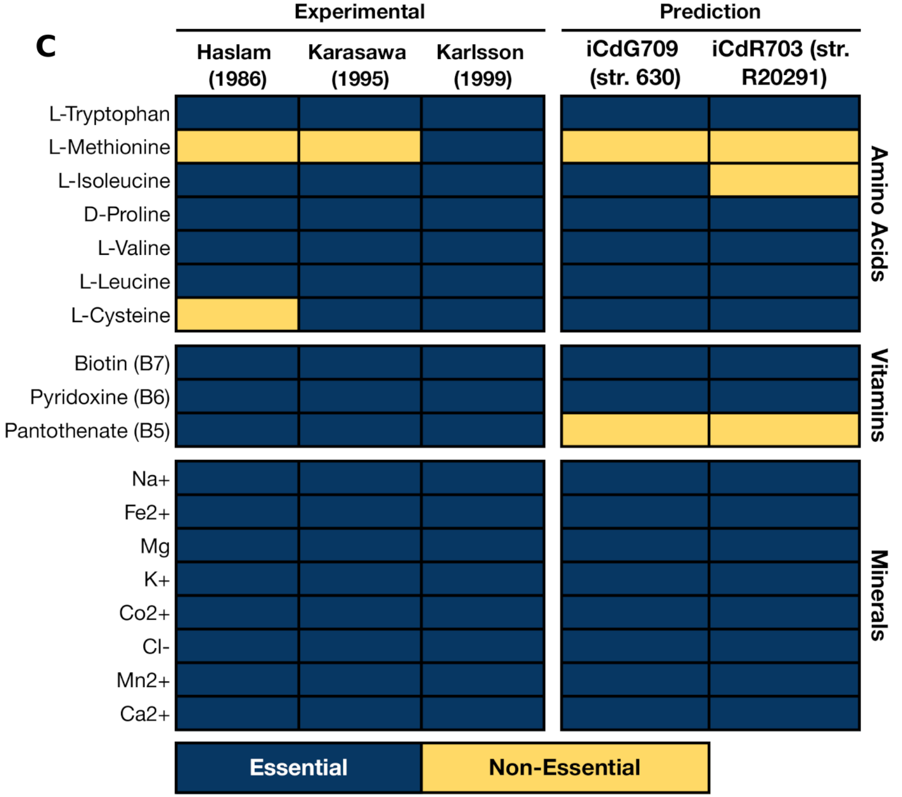

Supplement: FIG S1 [file msystems.00919-21-sf001.pdf]
