# Supplementary figures and images for: Novel Drivers of Virulence in Clostridioides difficile Identified via Context-Specific Metabolic Network Analysis
Source: mSystems. 2021 Oct 5;6(5):e00919-21. doi: 10.1128/mSystems.00919-21 (PMC8547418; doi:10.1128/mSystems.00919-21)

**A**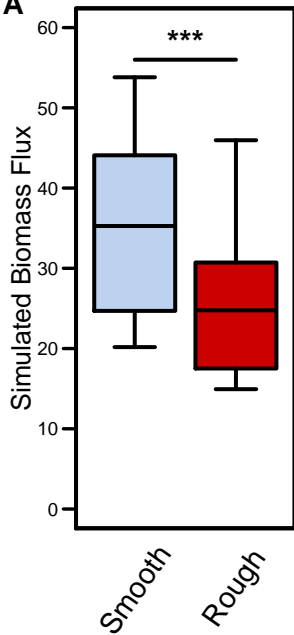**B**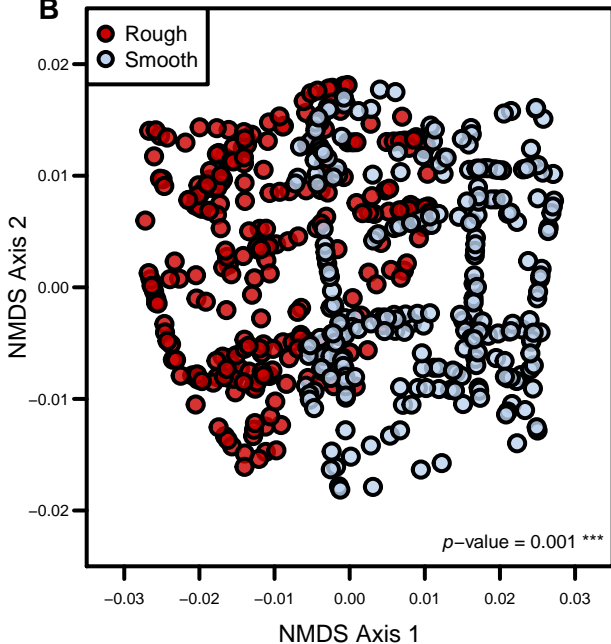

Supplement: FIG S2 [file msystems.00919-21-sf002.pdf]

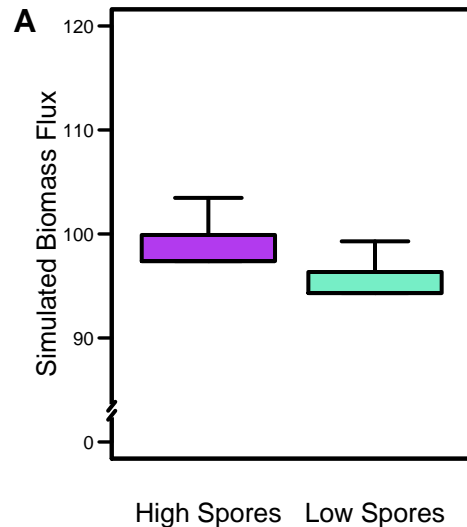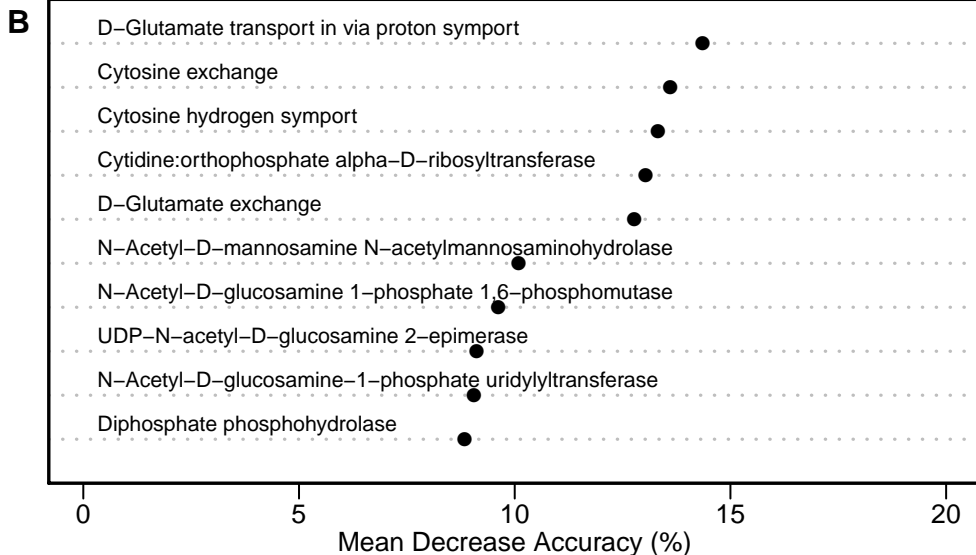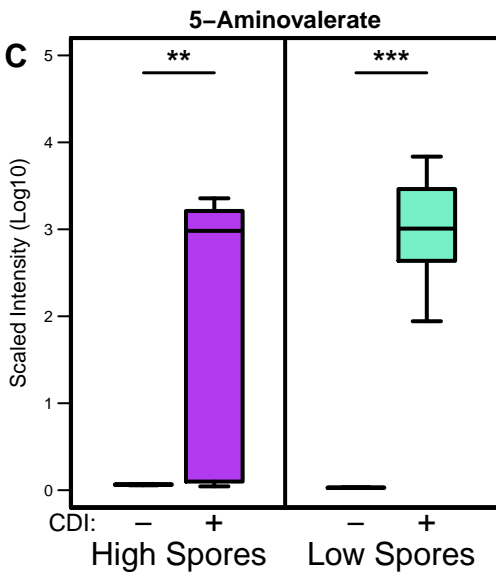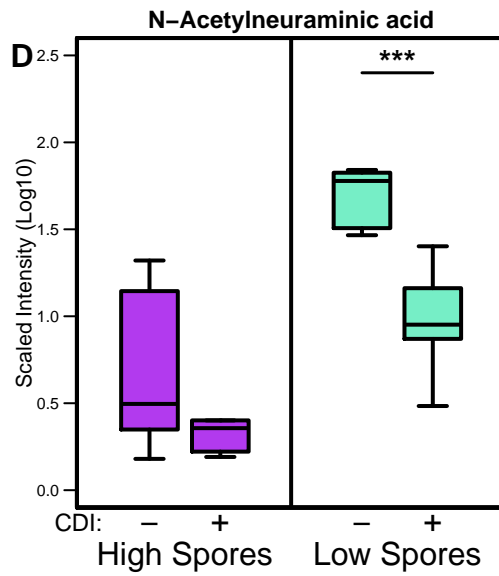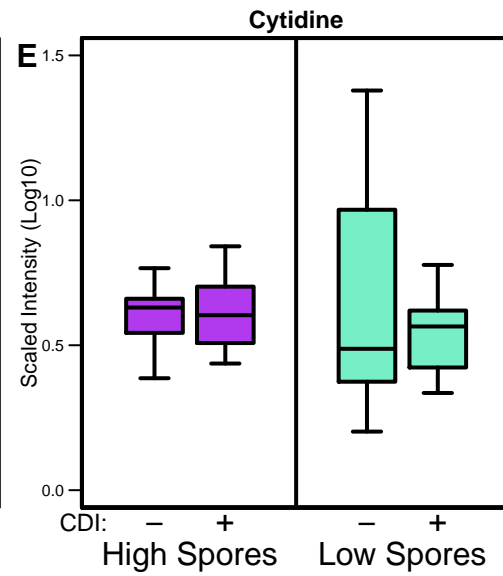

Supplement: FIG S4 [file msystems.00919-21-sf004.pdf]
