# Supplementary material for: Novel Drivers of Virulence in Clostridioides difficile Identified via Context-Specific Metabolic Network Analysis
Source: mSystems. 2021 Oct 5;6(5):e00919-21. doi: 10.1128/mSystems.00919-21 (PMC8547418; doi:10.1128/mSystems.00919-21)
Supplement: FIG S3 [file msystems.00919-21-sf003.pdf]

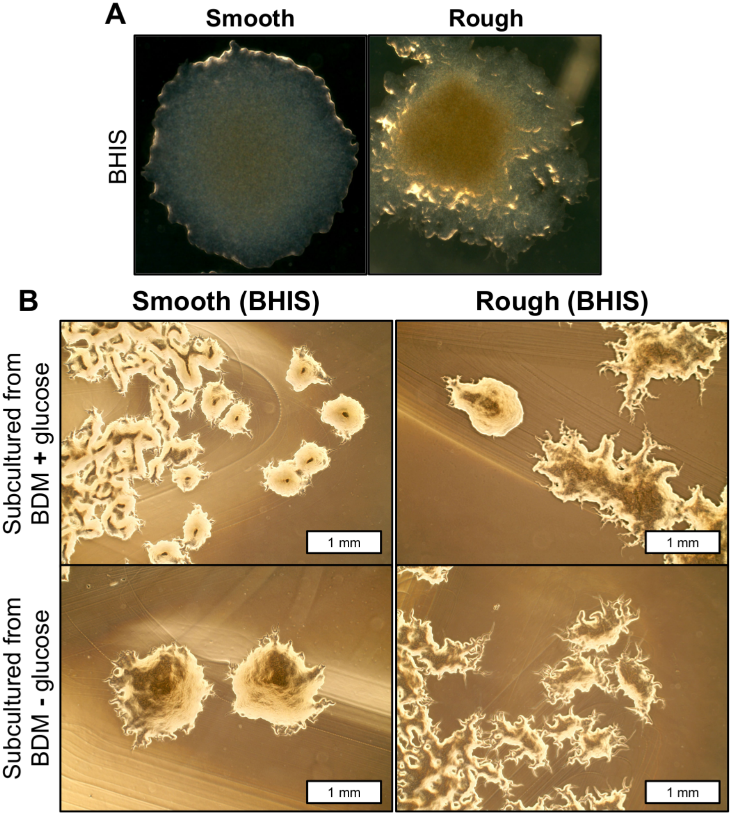
A
Smooth
Rough
BHIS
B
Smooth (BHIS)
Rough (BHIS)
Subcultured from BDM + glucose
Subcultured from BDM - glucose
1 mm
1 mm
1 mm
1 mm
