# Supplementary material for: Novel Drivers of Virulence in Clostridioides difficile Identified via Context-Specific Metabolic Network Analysis
Source: mSystems. 2021 Oct 5;6(5):e00919-21. doi: 10.1128/mSystems.00919-21 (PMC8547418; doi:10.1128/mSystems.00919-21)
Supplement: FIG S5 [file msystems.00919-21-sf005.pdf]

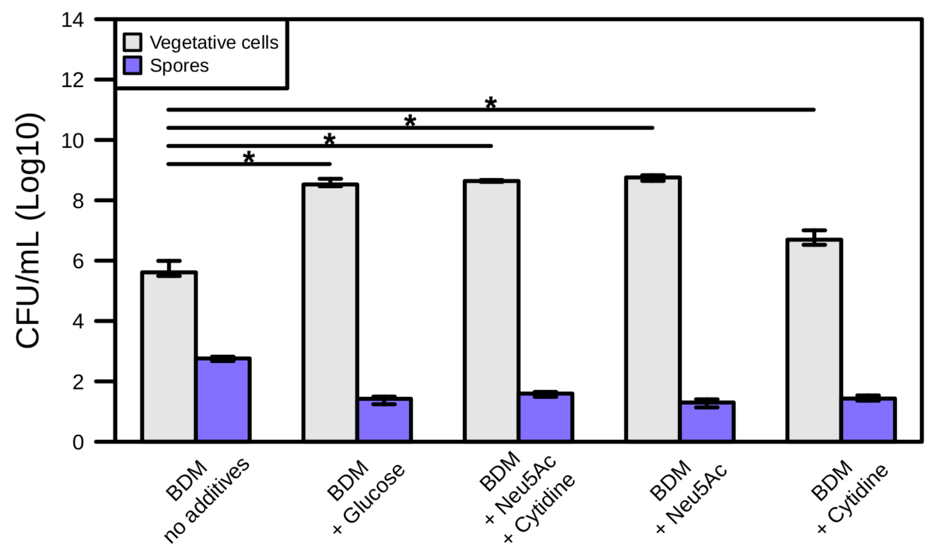
CFU/mL (Log10)
0
2
4
6
8
10
12
14
Vegetative cells
Spores
*
*
*
*
BDM no additives
BDM + Glucose
BDM + Neu5Ac + Cytidine
BDM + Neu5Ac
BDM + Cytidine
